# A DFT Study of Structural and Bonding Properties of Complexes Obtained from First-Row Transition Metal Chelation by 3-Alkyl-4-phenylacetylamino-4,5-dihydro-1H-1,2,4-triazol-5-one and Its Derivatives

**DOI:** 10.1155/2017/5237865

**Published:** 2017-07-03

**Authors:** Hubert Jean Nono, Désiré Bikélé Mama, Julius Numbonui Ghogomu, Elie Younang

**Affiliations:** ^1^Department of Chemistry, Faculty of Science, University of Dschang, P.O. Box 67, Dschang, Cameroon; ^2^Department of Chemistry, Faculty of Science, University of Douala, P.O. Box 24157, Douala, Cameroon; ^3^Department of Inorganic Chemistry, Faculty of Science, University of Yaoundé I, P.O. Box 812, Yaoundé, Cameroon

## Abstract

Density functional calculations were used to explore the complexation of 3-alkyl-4-phenylacetylamino-4,5-dihydro-1h-1,2,4-triazol-5-one (ADPHT) derivatives by first-row transition metal cations. Neutral ADPHT ligand and mono deprotonated ligands have been used. Geometry optimizations have been performed in gas-phase and solution-phase (water, benzene, and N,N-dimethylformamide (DMF)) with B3LYP/Mixed I (LanL2DZ for metal atom and 6-31+G(d,p) for C, N, O, and H atoms) and with B3LYP/Mixed II (6-31G(d) for metal atom and 6-31+G(d,p) for C, N, O, and H atoms) especially in the gas-phase. Single points have also been carried out at CCSD(T) level. The B3LYP/Mixed I method was used to calculate thermodynamic energies (energies, enthalpies, and Gibb energies) of the formation of the complexes analyzed. The B3LYP/Mixed I complexation energies in the gas phase are therefore compared to those obtained using B3LYP/Mixed II and CCSD(T) calculations. Our results pointed out that the deprotonation of the ligand increases the binding affinity independently of the metal cation used. The topological parameters yielded from Quantum Theory of Atom in Molecules (QTAIM) indicate that metal-ligand bonds are partly covalent. The significant reduction of the proton affinity (PA) observed when passing from ligands to complexes in gas-phase confirms the notable enhancement of antioxidant activities of neutral ligands.

## 1. Introduction

In recent years, the repercussion of free radicals and reactive oxygen species (ROS) in neurodegenerative disorder is more sensitive [1–4]. The contribution of these ROS to the pathophysiology of myocardial reperfusion damage. These ROS can be oxygen-centered radicals [5] or oxygen-centered nonradicals [6]. The removal of electrons from cellular membranes by these ROS and the reaction between these latter ones and proteins [7] provoke the alteration of the structures of these membranes and proteins. Such alterations justify the frailty of these cellular membranes that expose them to be attacked by invaders (viruses and bacteria).

Nevertheless, each cell is naturally equipped by defense systems against any destructive effect of ROS. This statute of protective mechanism against ROS in humans is attributed to antioxidant molecules [8, 9]. In general, antioxidant molecules (tocopherol (vitamin E), ascorbic acid (vitamin C), carotenoids, flavonoids, and polyphenols) prevent the proliferation of free radical reactions in all cell membranes. This explains the emergence of studies on the investigation of antioxidant activities of biologically active compounds. This has led to increased pressure on the need for the newer molecules which may have potentials to curb the spread of this problem. Particularly, 1,2,4-triazole and its derivatives have been reported to possess antioxidant activities [10–13]. The 1,2,4-triazole derivatives have also been known to possess many biological activities (antifungal, analgesic, antiviral, anti-inflammatory, antitumor, anti-HIV properties, etc.) [14, 15]. It is worthwhile to mention that 1,2,4-triazoles have been prepared by different methods. The cyclodehydration of acylthiosemicarbazides with a variety of basic agents is the most common method used. The literature survey revealed that acylthiosemicarbazides are the key intermediates used in the synthesis of 1,2,4-triazol [16, 17].

Our previous research was focused on the theoretical analysis of antioxidant mechanisms of 1,2,4-triazole derivatives [18], more precisely of 3-alkyl-4-phenylacetylamino-4,5-dihydro-1H-1,2,4-triazol-5-one (ADPHT) derivatives. The calculated thermodynamic properties descriptors calculated in gas and solution-phases were Hydrogen Atom Transfer (HAT), Single Electron Transfer-Proton Transfer (SET-PT), and Sequential Proton-Loss Electron Transfer (SPLET) mechanism. Results indicated that, thermodynamically, HAT mechanism is the most predominant process in the gas-phase. But, in solvents (2-propanol, acetonitrile, DMF and water), the SPLET mechanism has shown to be more preferred. It has been shown that the removal of the metals by metal-chelating process causes some problems of toxicity due to the induced charge change [19]. But the examination of the literature demonstrates that the transition metal chelation of the titled ligands has not been done either experimentally or theoretically.

The goal of this work is to do a comprehensive density functional theory (DFT) study on the first-row transition metal (II) chelation by ADPHT derivatives, using both the neutral and mono deprotonated forms of ligands. We have studied the coordination abilities for the first-row transition metal (II) cations used (Fe^2+^, Ni^2+^, Cu^2+^, and Zn^2+^). The authors evaluated the proton affinity (PA) of each complex and therefore analyzed the comparative impact of the metal chelation on the antioxidant activities. The proton affinity free energy (PAFE) has also been taken into account. The metal interaction is further studied on the basis of NBO charges. The replacement of M^2+^ by the M^+^ leads to the evaluation of the influence of the metal charge on analyzed properties. For this last point, the authors have considered only the copper atom as metal. Estimation of the effect of solvents (water, benzene, and N,N-dimethylformamide (DMF)) on the calculated structural parameters has also been done.

## 2. Computational Details and Theoretical Background

### 2.1. Computational Details

All calculations were performed using Gaussian 09 program package [20]. The optimization of gas-phase structure of each molecular system was obtained using DFT method with B3LYP [21] functional. For M^2+^ (M^2+^ = Fe^2+^, Ni^2+^, Cu^2+^, and Zn^2+^), the authors used the nonrelativistic effective core potential (ECP) LanL2DZ [22] for metal atom in combination with 6-31+G(d,p) for C, N, O, and H atoms. This generically made basis set is denoted as Mixed I. The importance of using ECPs for transition metal complexes has been emphasized by prior researches [23, 24]. The geometry optimization is followed by vibrational frequency calculations. All of these calculations were carried out in vacuum and in three solvents (water, benzene, and DMF (N,N-dimethylformamide)). The integral equation formalism of the polarized continuum [25, 26] has been taken into account to analyze the solvent effect. To evaluate the influence of the basis set on geometries, an additional geometrical optimization of the gas-phase structures followed by frequency calculations using a new mixed basis set [M^2+^ (Fe^2+^, Ni^2+^, Cu^2+^, and Zn^2+^) = 6-31G(d) and 6-31+G(d,p) = C, N, O, and H] subsequently denoted as Mixed II has been achieved.

### 2.2. Theoretical Background

#### 2.2.1. Thermodynamic Energies of Coordination Abilities and Deprotonation of Complexes

The coordination ability of various cations (*D*_*e*_) (dissociation energy of the complex noted *c*) is defined according to (1):

(1) $$\begin{array}{c} D_{e}=E_{c}-\left(\;E_{l}+E_{m}\;\right). \end{array}$$

*E*_*c*_, *E*_*l*_, and *E*_*m*_ are, respectively, the energy of the complex, ligand, and metal. Firstly, *D*_*e*_ values are estimated after geometrical optimization calculations at B3LYP/Mixed I in the gas-phase and in various solvents and reevaluated at B3LYP/Mixed II level in the gas-phase Single-point calculations at the CCSD (T) have been carried out to reproduce the gas-phase *D*_*e*_. The levels of theory have shown to reproduce satisfactorily the first-row transition metal binding energy of glycine and its derivatives [27, 28]. The Metal Ion Affinity (MIA) [29, 30] was assumed to be the negative of the reaction enthalpy (Δ*H*_298_^0^) defined in (2). We have determined the complexation free energy according to (3):

(2) MIA=−ΔH2980=−H2980c−H2980l+H2980m

(3) ΔG2980=G2980c−G2980l+G2980m.

#### 2.2.2. NBO Analysis

The stabilization energy *E*_2_ associated with *i* → *j* delocalization for a donor NBO (*i*) and an acceptor NBO (*j*) is calculated according to

(4) $$\begin{array}{c} E_{2}=q_{ij}\frac{F^{2}\left(i,j\right)}{\varepsilon_{j}-\varepsilon_{i}}, \end{array}$$

where *q*_*ij*_ represents the *i*th donor orbital occupancy and *ε*_*i*_ and *ε*_*j*_ are diagonal elements and *F*(*i*, *j*) off-diagonal elements, respectively, linked to NBO Fock matrix [31].

The thermodynamic energies (proton affinity (PA) and proton affinity fee energy (PAFE)) relative to the deprotonation of each optimized complex are determined as follows:

(5) MLHn+⟶MLn−1++H+

(6) PA=HH++HMLn−1+−HMLHn+

(7) PAFE=GH++GffMLn−1+−GMLHn+.

[*MLH*], [*ML*]^+^, and *H*^+^ are, respectively, complex and deprotonated complexes and the proton. *H*(*Y*) is the enthalpy of species *Y*  (*Y* = *MLH*, *ML*^+^, and *H*^+^). Solvent contribution was determined using an integral equation continuum model (IEF-PCM) method [25, 26].

#### 2.2.3. Atoms in Molecules Analysis Theory

The Quantum Theory of Atom in Molecules (QTAIM) proposed by Bader [32] performed as implemented in Multiwfn [33] was to analyze the nature of all metal-ligand bonds. A logical approximation of their relative energies eases the specification of the original nature of metal-ligand bonds. The indicators of metal-ligand bond used are the electron densities *ρ*(*r*) and their Laplacians ∇^2^*ρ*(*r*) calculated at the bond critical points (BCPs). The local kinetic electron energy density and the potential energy density *ν*(*r*) are then defined, respectively, in (8) and (9) [32, 34]. Consider

(8) Gr=3103π2/3ρr5/3+16∇2ρr

(9) νr=ħ24m∇2ρr−2Gr.

According to the sign of the Laplacian of the electron density (∇^2^*ρ*(*r*)), the metal-ligand interactions are covalent and electrostatic, if ∇^2^*ρ*(*r*) are, respectively, negative and positive. A further instrument for estimation of the nature of the metal-ligand is the fraction −*G*(*r*)/*ν*(*r*). From this descriptor, the metal-ligand bond is noncovalent or partly covalent in nature, if −*G*(*r*)/*ν*(*r*) > 1 and 0.5 < −*G*(*r*)/*ν*(*r*) < 1, respectively. This ration combined with the electron density (∇^2^*ρ*(*r*)) is useful to analyze the intermediate interactions and closed shell interaction: (−*G*(*r*)/*ν*(*r*) < 1  and  ∇^2^*ρ*(*r*) < 0)) and (−*G*(*r*)/*ν*(*r*) > 1 and ∇^2^*ρ*(*r*) < 0)), respectively.

This analysis is using B3LYP/Mixed II optimized structures in the gas-phase. QTAIM analysis was then performed as implemented in Multiwfn [32].

## 3. Results and Discussion

**Figure 1:**
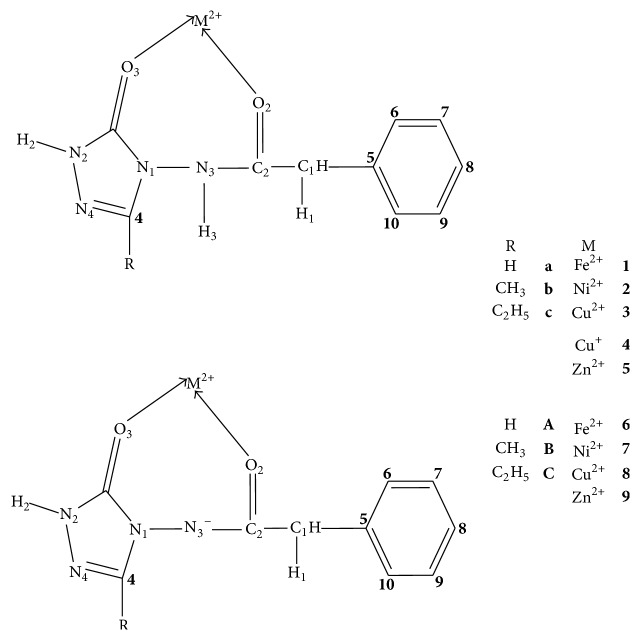
Schematic representation of M-ADPHT complexes including the adopted numbering system used.

We have optimized the M-ADPHT complexes using all the possible coordination modes. These optimizations yield a unique coordination mode (O_3_, O_2_) as shown in Figure 1 for neutral or deprotonated ADPHT derivatives.

### 3.1. Geometrical Details

All the structures have been optimized without any symmetric constraint. The relevant geometrical parameters of M-ADPHT complexes with neutral ligands, labeled according to convention given in Figure 1, are compiled in Table 1. The optimization generally yields M-O_2_ bond distances longer, compared to those of M-O_3_ bonds. The bond length difference was in the following range: 0.009–0.077 Å. We attributed such a difference to the fact that the C_3_=O_3_ carbonyl group is connected to two N_1_ and N_2_ nitrogen atoms. The induced cumulative electron donating effect of these two neighboring nitrogen atoms then increases the electron density around the O_3_ oxygen atom. This atom is consequently more nucleophile than the O_2_ homolog which is near only one N_3_ nitrogen atom. Our results showed that the M-O_*i*_ (*i* = 2, 3) decreased slightly with the substitution of the hydrogen by donor alkyl group (R = CH_3_, C_2_H_5_). Independently of the substituent, the Cu-O_*i*_ (*i* = 2, 3) distances are the longest in gas-phase. The replacement of Cu^2+^ (d^9^) by Cu^+^ (d^10^) leads to the shortening of Cu-O_2_ bond lengths but also to the lengthening of Cu-O_3_ bond distances. This fact originates predominantly from the interaction between the doubly occupied metal 3d orbital and the lone pairs of donor oxygen atoms, which significantly increases the metal-ligand repulsion in the Cu^+^ (d^10^)-complexes. This does not occur for Cu^2+^ (d^9^) for which the 3d orbital remains singly occupied. Our results on Cu^+^ (d^10^)-complexes are in line with similar theoretical works on monovalent metal cation (Co^+^, Ni^+^, and Cu^+^)-ligands (glycine [35], water [35, 36], ammonia [37, 38], and adenine [39]) complexes. We concluded that this metal-ligand repulsion in Cu^+^ (d^10^)-complexes is more pronounced for the more nucleophile oxygen atom O_3_. The comparison of the bond distances of Cu^+^-complexes with those of Cu^2^-complexes (Table 1) reveals the fact that the effects of this substitution are only limited to metal-ligand bond distances. The dissimilarity observed between distances of bonds of ADPHT complexes and those of isolated ligand displays the significant activation of adjacent bonds by the metal cation. For instance, the metal chelation of ligand** a** by Cu^2+^ augments the C_3_-O_3_ bond distance from 1.216 to 1.250 Å.

The optimized O_2_-M^2+^-O_3_ angle in all M^2+^-complexes in gas-phase varies from 98.3 to 106.4°. The dihedral angles Φ [N_3_-C_2_-O_2_-M^2+^] and Φ [N_1_-N_3_-C_2_-O_2_] were, respectively, in the following range: −57.3–59.1 and −35.6–53.3°. This fact renders chelate rings of M-ADPHT (M = Fe^2+^, Ni^2+^, and Cu^2+^) nonplanar. In addition, the values obtained for the dihedral angles Φ [N_1_-N_3_-C_2_-O_2_] revealed that the N_1_, N_3_, C_2_, and O_2_ atoms are not located in the same plane (Table 1). Our B3LYP data revealed that the benzene and 1,2,4-triazole ring remained planar (Figure 1S) in the supporting information, available online at https://doi.org/10.1155/2017/5237865). In the same vein, the calculated values of the dihedral angle Φ [N_3_-C_2_-C_1_-C_5_] indicated the fact that the C_5_ atom is almost in the plane containing N3, C_2_, and C1 atoms, which is nearly perpendicular to the benzene ring.

Contrary to the results obtained in gas-phase, the optimization after the solvation of the molecular system yields M-O_3_ bond distances longer, compared to those of M-O_2_ bonds especially for complex 1a in benzene and DMF. One can observe that the variations of the geometrical parameters are very versatile in solution-phase.

**Figure 2:**
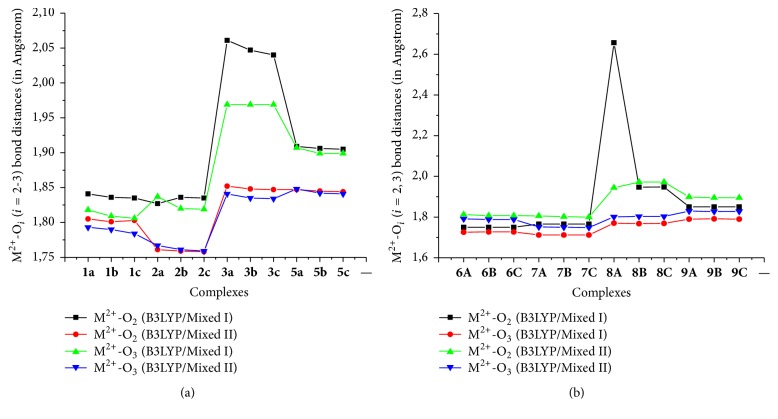
Superposition of M^2+^-O_*i*_ bond obtained using B3LYP/Mixed I, B3LYP/Mixed II, and CCSD(T) for neutral ADPHT ligands (a) and for deprotonated ADPHT ligands (b).

**Figure 3:**
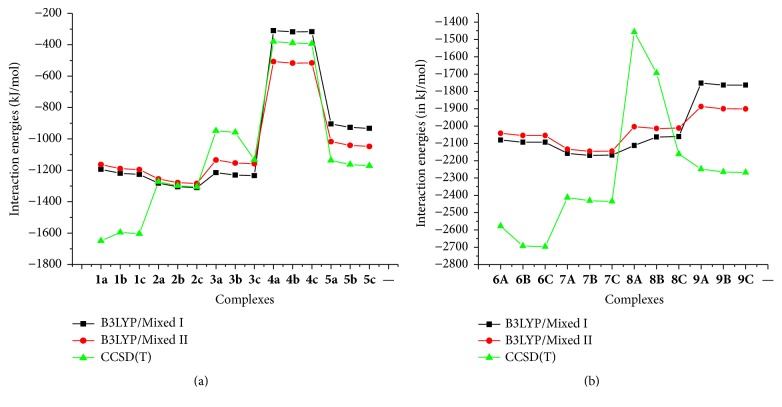
Superposition of metal binding energy values (in kJ/mol) obtained using B3LYP/Mixed I, B3LYP/Mixed II, and CCSD(T) for neutral ADPHT ligands (a) and for deprotonated ADPHT ligands (b).

To investigate the contribution of the metal chelation to the antioxidant activity of ADPHT derivatives, we analyzed the X-H bond distances (X = N_3_, N_2_, and C_1_) of the structures obtained and compared them with those of isolated ligands. From Figure 2S, one can find out that the metal chelation slightly increases the X-H bond distances, then decreases the bond dissociation energies of these bonds, and therefore enhances the antioxidant activities. In M-ADPHT complexes optimized, the longer X-H bond distances are obtained for C_1_-H_1_ bonds due to the captodative stabilization evoked in our previous work [18]. Figure 3S displays the fact that the solvation of complexes induces an important reduction of the C_1_-H_1_ bond distances. Such a reduction is more pronounced for Cu^2+^-complexes in benzene and DMF.

Geometrical parameters of optimized complexes obtained in gas-phase from deprotonated ligands (Tables 1–3S in the supporting information) illustrated the fact that the metal-ligand distances are lower than those of complexes resulting from neutral ligand, with the exception of 8A complex. The main justification of this exception resulted from the optimization of 8A complex that leads to a monodental structure with the O_3_ oxygen only effectively binded to the Cu^2+^ cation. Our results also showed that the M-O_3_ bond distances are slightly longer than those of M-O_2_ bonds. This is basically due to the nearness of the C_2_=O_2_ carbonyl group to proton abstraction site (N_3_ atom) that subsequently increases the electron density around the O_2_ oxygen atom. Contrary to previous remarks on complexes obtained from neutral ligands, the latter becomes now more nucleophile than the O_3_ oxygen atom.

Figure 2 indicates that the M^2+^-O_*i*_ (*i* = 2,3) bond distances yielded by B3LYP/Mixed II are shorter than that relative to B3LYP/Mixed I. The bond distance differences obtained are in the ranges 0.023–0.887 and 0.019–0.169 Ǻ, respectively, for M^2+^-O_2_ and M^2+^-O_3_ bond. This fact can be attributed to the fact that the valence orbitals are not properly described by B3LYP/Mixed I. This results from the poor description of the electron-electron repulsion. In the whole, higher differences are obtained for Cu^2+^-complexes for both ligands.

### 3.2. Metal Binding Selectivity

Table 2 gives the B3LYP/Mixed I complexation energies of the M-ADPHT complexes in various media. All the complexation energies are highly negative showing that the reaction is highly exothermic. One can observe a relevant decrease of the binding energies when passing from the gas-phase to solvent-phases. This drop is directly attributed to solvent effect that hampers the interaction between transition metal cation and ADPHT ligand. This diminution is more pronounced for protic solvents (water and DMF).

The complexation energies for solvent-phase are correlated to M-O_*i*_ (*i* = 2, 3) bond distances presented in the previous section.  Figure 3 and Table 2 display for neutral ligands (**a**,** b,** and** c**) an increasing binding selectivity in the following order: Zn^2+^ < Fe^2+^ < Cu^2+^ < Ni^2+^. This is in line with the previous works which exhibited the highest affinity of Ni^2+^ compared to other divalent first-row transition metal cations for glycine, glycine derivatives [40], polyether [41], and polyamine ligand [41]. The M^2+^-ADPHT complexes have higher metal ion affinities than the Cu^+^-ADPHT complexes. For both complexes, the higher binding abilities are obtained for c neutral ligand.

Our data show that the deprotonation of the ligand increases the binding affinity independently of the metal cation used. From Table 2, it is revealed that the binding energy difference between the value obtained from neutral ligand and that from deprotonated ones is in the following ranges: 827–897 kj/mol for M^2+^-ADPHT and 438–447 kj/mol for Cu^+^-ADPHT in gas-phase. The calculated trend for *D*_*e*_ is in agreement with the notable decrease of M-O_*i*_ (*i* = 2, 3) bond distances of optimized complexes obtained in gas-phase from deprotonated ligands evoked in previous section (Figure 2 and Tables 1–3S in the supporting information). This increase of binding affinity is also observed in solution-phase. For the deprotonated ligand A, a similar increasing binding selectivity is observed compared to that of neutral ligand, whereas Figure 3 clearly reveals that the B3LYP/Mixed I complexation energies of the divalent ion with deprotonated ligands (B and C) follow the order of Zn^2+^ < Cu^2+^ < Fe^2+^ < Ni^2+^. A similar situation was found for B3LYP/Mixed II values. In the whole, the latter is higher than the former for divalent transition cations with the exception of Zn^2+^-complexes. The average difference reached 6.2 and 67.0 kJ/mol, respectively, for neutral ADPHT –M^2+^ complexes and deprotonated ADPHT –M^2+^ complexes. For neutral ADPHT –Cu^+^ complexes, Figure 3 also displays the drastic drop that is in 197.3–199.7 kJ/mol. In the same vein, difference in complexation energy values in the range 100.5–641.4 kJ is observed for deprotonated ADPHT –M^2+^ complexes when passing from B3LYP calculations to CCSD(T) ones (Figure 3). This similar larger difference has been found by Constantino et al. in the theoretical study on interactions of Co^+^ and Co^2+^ with glycine [35]. We attributed this fact to a bad annulment of self-interaction term by the exchange functional that leads to overstabilization of molecular systems by density functional methods. For neutral ADPHT –M^2+^ complexes, this difference is variable. In the case of Ni^2+^-complexes, differences are in a very narrow range of 4.4–9.5 kJ/mol for calculations related to B3LYP/Mixed II.

The calculated interaction enthalpies presented in Table 2 do not give any additional relevant information on the metal binding selectivity. In order to get a deep insight into the capacity of ADPHT ligands to be bound to metal cations, we have calculated the interaction free energies. The calculated results obtained in various media inserted in Table 2 are negative showing that the formation of M^n+^-ADPHT complexes (*n* = 1, 2) is spontaneous. A comparison of the interaction free energies of Cu^2+^ and Cu^+^ in various media shows that the preference of both metal cations depends on the nature of the ADPHT ligands.

We note that higher capacity of the neutral ligand to bind to Cu^+^ cation is observed. Contrary to monovalent copper cation, higher capacity is attributed to complexation of deprotonated ligand to Cu^2+^. We find again that our results indicate that all divalent metal cations prefer to be bound to deprotonated ADPHT ligands. In gas-phase, the lower interaction free energies obtained for Ni^2+^-ADPHT complexes confirms the preference of both ligands to bind to Ni^2+^ previously evoked in this section.

### 3.3. Electronic Structure and Atoms in Molecules Analysis

**Figure 4:**
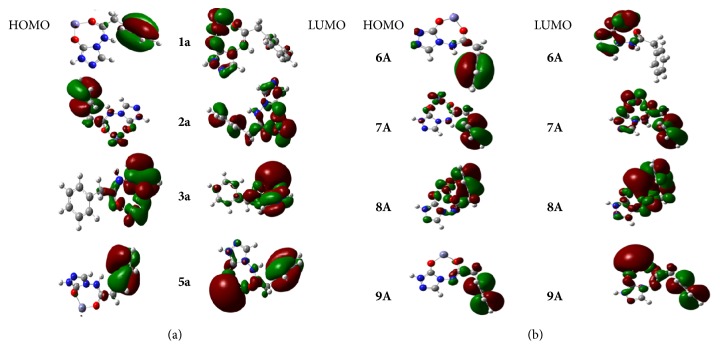
Schematic representation of frontier molecular orbitals of M-ADPHT complexes (R = H): neutral ligand-divalent metal cation complexes (a) and deprotonated ligand-divalent metal cation complexes (b).

In previous studies, it has been found that the HOMO-LUMO energy gap is an important stability descriptor [41–44]. The large HOMO-LUMO energy gap is consistent to stable and little reactive systems, but, in the contradictory case, the little stable systems correspond to highly reactive systems. The orbital frontier eigenvalues and HOMO-LUMO gaps of different complexes calculated in various media are inserted in Table 3. Our data reveal that deprotonated ADPHT ligand-M^2+^ complexes are the most stable ones in gas-phase. The highest HOMO-LUMO energy gap matches up with the monodental complex 8A in the same medium. This explains why the electron cloud of HOMO is mainly localized on atoms of benzyl rings and on the metal atom for complex 8A (see Figure 4). On the contrary, for other complexes, this cloud is exclusively located on atoms of benzyl ring. The solvation studied enhances the stability of the complexes studied. This enhancement is more pronounced for protic solvent (water and DMF). The comparison between the HOMO-LUMO gap on isolated ligands (Table 4S in the supporting information) and that of complexes reveals that the metal chelation of isolated ligands reduces their stabilities independently of the medium.

The HOMO eigenvalues are used to characterize the donating ability of the molecule. Higher value of HOMO energy indicates a predisposition of the molecular system to loss electrons [45]. Higher value of HOMO energy is therefore an indication of the higher antioxidant activity [46].

In gas-phase, the values of HOMO energy of the isolated ligands (Table 4S in the supporting information) exhibit *E*_c_ > *E*_b_ > *E*_a_ for neutral ligands and *E*_B_ > *E*_C_ > *E*_A_ for deprotonated ones. One can then conclude that the orders for the antioxidant activity of these ADPHT ligands are c > b > a and B > C > A, respectively, for neutral ligands and deprotonated homologs. For the former, this order remains invariable upon chelation with a metal ion with exception made to Ni^2+^ (c = b > a) in gas-phase. For the latter, the complexation of the deprotonated ligand leads to versatile results. This result corroborates with previous theoretical research on Hydrogen Atom Transfer in the reaction of metal-associated phenolic acids with OH^∙^ radical that showed that the ordering for antioxidant activity of the neutral phenolic acids does not change upon chelation with a divalent metal cation [45]. Our results also demonstrate that the solvation of complexes augments the antioxidant activity of the complexes. This augmentation is more sensitive for protic solvents (water and DMF). These results are consistent with previous studies on neutral ADPHT ligand [18] and other molecular systems [47, 48]. The explanation is that the charge-separation process is quite sensible to polarity of solvent [49]. From Table 3, an increase of dipole of complexes is observed with the increase of the solvent's polarity declines the strength of X-H (X = N, C) bonds and subsequently increases the antioxidant activity of complexes formed. This conclusion is in line with previous works [47–51]. The survey of HOMO eigenvalues and dipole moments of optimized isolated ADPHT ligands (Table 4S in the supporting information) compared to those of its complexes highlights the augmentation of antioxidant activity at the end of the complexation in various media.

Table 3 also shows an authentication of charge donation from ligand to metal atom. This donation is more sensitive for deprotonated ligands. Such a greater electron transfer from these ligands to metal (II) atom is a plausible explanation of their higher stabilities previously evoked. Our data also expose a greater electron transfer Cu(II)-complexes for both ligands. The calculated NBO charges on metal (II) atom are in good agreement with previous theoretical researches [40, 52] that disclosed the fact that greater electron transfer from the electron donor to the acceptor leads to higher stability. On the whole, the value of the metal charge carried by the metal cation in gas-phase was in the following ranges: +(0.81–1.23) for iron, +(0.95–1.04)*e* for nickel, +(0.75–0.92)*e* for copper, and +(0.9–1.61)*e* for zinc. This shows that a real charge transfer leads to an increase of the electronegativity of the metal ions that then has undergone a reduction. We then concluded that the metal cation plays an oxidation role towards both ligands. These observations are similar to those made on quercetin [53] and on phenolic acids [52].

**Figure 5:**
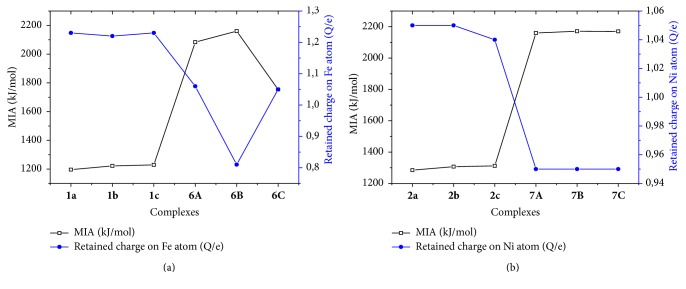
Correlation between the MIA (kJ/mol) and retained charge (Q/e) of Fe (a) and correlation between the MIA (kJ/mol) and retained charge (Q/e) of Ni (b).

**Figure 6:**
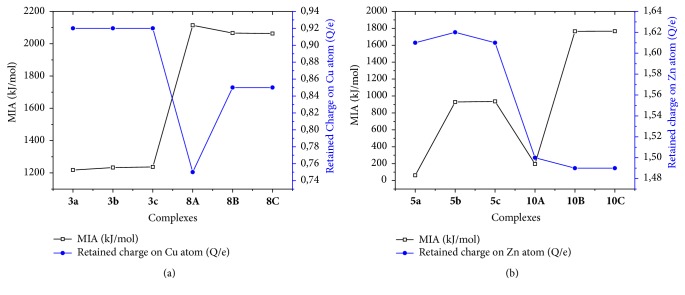
Correlation between the MIA (kJ/mol) and retained charge (Q/e) of Cu (a) and correlation between the MIA (kJ/mol) and retained charge (Q/e) of Zn.

To see the possible correlation between the retained NBO charge on the metal atom and metal MIA of each of the metal atoms, the two parameters are plotted (Figures 5 and 6). In the four cases (Fe^2+^, Ni^2+^, Cu^2+^, and Zn^2+^), MIA values vary proportionately with the retained charge of the metal ion for deprotonated ADPHT ligands. On the contrary, MIA values vary inversely with the retained charge of the metal cation. Our results show that the solvation diminishes the electron donation from the ligand to metal atom, except for 5*i* (*i* = a, b, and c) complexes in water and for 8C in benzene.

The differences in retained charge on metal atom resulting from the various media were in the following ranges: (0.15–0.46)*e* for water, (0.05–0.43)*e* for benzene, and (0.14–0.59)*e* for DMF (see Table 3). Therefore, one could presume that the diminution of electron transfer is more enunciated in polar solvent.

**Figure 7:**
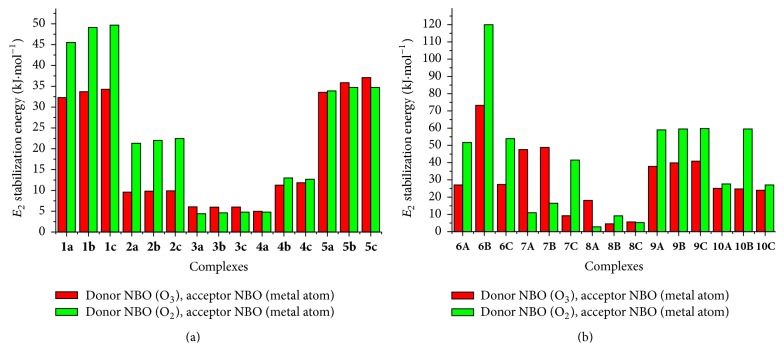
Second perturbative interaction energies *E*_2_ (in kJ·mol^−1^) between the first-row transition metals and lone pair electron ligand atoms (oxygen atom O_3_ and oxygen atom O_2_) for each complex: *E*_2_ energies for neutral ligands (a) and *E*_2_ energies for deprotonated ligands (b).

So as to get detailed information on electron transfer in the coordination sphere complex, we presented in Figure 7 the second-order perturbation energy stabilization (*E*_2_) associated with the electron donation: electron donor (O_2_ or O_3_) to the acceptor (metal atom). The *E*_2_ values range from 4.4 to 49.68 kJ/mol and from 2.76 to 119.98 kJ/mol, respectively, for metal-neutral ligand complexes and metal-deprotonated ligand complexes. The higher gap between the contribution of O_2_ → metal donation and that of O_3_ → metal ones is obtained for Fe-complexes in both cases (with exception to 6C) in favor of the former one. This higher gap varies from 12.24 to 46.78 kJ/mol. This higher contribution of O_2_ → metal donation is also observed when the Cu^+^ and Zn^2+^ were chelated to the deprotonated ligands.  Figure 6 also exhibits a drastic drop of both contributions for Cu^2+^-complexes. The negligible contribution of O_2_ → metal donation (2,76 kJ/mol) highlighted the monodental nature of 8A structure evoked in the prior geometrical analysis. To better appreciate the impact of the metal chelation beyond the coordination inner-sphere, we made a comparison between calculated energies of hyperconjugative interaction for neutral ADPHT ligands and those of its complexes (Table 5S in supporting information). In the whole, this comparison exhibits the noteworthy influence of the metal chelation on the interaction within both ligands used in this work. This fact is in agreement with the significant activation of adjacent bonds of metal cations previously underlined.

The topological parameters obtained at B3LYP/Mixed II level are inserted in Tables 4 and 5. The positive ∇^2^(*ρ*(*r*)) values and negative *H*(*r*) values obtained illustrate that the M^2+^-O_i_ (*i* = 2,3) bonds are partly covalent. This result is confirmed by the fact 0.5 < −*G*(*r*)/*ν*(*r*) < 1. Close inspection of Table 4 reveals that the *ρ*(*r*) values of M^2+^-O_3_ bond are larger than that for M^2+^-O_2_ bond for M^2+^-neutral ADPHT complexes with exception of** 2a**. This observation points out that M^2+^-O_3_ bonds are stronger than M^2+^-O_2_ bond of these complexes. This corroborates the fact that M^2+^-O_2_ bonds are stronger than M^2+^-O_2_ bonds. Nevertheless, Table 4S displays contrary information for M^2+^-deprotonated ADPHT complexes. The *ρ*(*r*) values of M^2+^-O_*i*_ (*i* = 2,3) in Cu^2+^-deprotonated ADPHT complexes are higher than that for Cu^+^ complexes. This confirms the preference of Cu^2+^ to bind deprotonated ligand. The *ρ*(*r*) values of M^2+^-O_*i*_ (*i* = 2,3) in M^2+^-deprotonated ADPHT complexes are sensitively higher than its homolog in M^2+^-neutral ADPHT complexes with exception to Fe^2+^-complexes. The *ρ*(*r*) value difference ranges from 0.006 to 0.038. Consequently, the M^2+^-O_i_ (*i* = 2,3) in M^2+^-deprotonated ADPHT complexes are stronger than that in M^2+^-neutral ADPHT complexes. This result is in line with the fact that metal ligand distances in the former are lower.

### 3.4. Proton Affinity

In order to investigate the possibility of deprotonation of metal-neutral ligand complexes in various media, the authors analyzed the PA and PAFE values of the complexes. We absolutely need the enthalpy and free energy of H^+^ to determine the thermodynamic energies. The calculated values of these thermodynamic energies used in this work are compiled in Table 6S. In the interest of comparison, we presented in Table 7S the PA and PAFE values of isolated neutral ligands and its complexes in various media. A major drop of the PA values is observed when passing from ligands to complexes in gas-phase. This drop is lower for Cu^+^-complexes.

Such a drop discloses the notable enhancement of antioxidant activities of neutral ligands in gas-phase. This fact is in good agreement with the increase of the X-H bond distances induced by the metal chelation (shown in Figure 1S). This augmentation of the antioxidant activities has been pointed out in our previous researches [54]. The difference in PA values from ligands to complexes declines in solution-phase.

We therefore concluded that the solvation reduces the enhancement of the antioxidant activities induced by the metal chelation. The positive PA values obtained for all the complexes revealed that the deprotonation of metal-neutral ligand complexes is endothermic in gas-phase, water, and DMF (3a complex excepted). It is fair to note that this deprotonation remains endothermic in benzene only for Cu^+^ and Zn^2+^-complexes. The positive PAFE values obtained show that the deprotonation of metal-neutral ligand complexes is not spontaneous with exception made to minority of endothermic deprotonation previously mentioned.

## 4. Conclusions

In this study, we have presented the B3LYP/Mixed I and B3LYP/Mixed II calculations which allowed us to treat the complexation of 3-alkyl-4-phenylacettylamino-4,5-dihydro-1h-1,2,4-triazol-5-one derivatives by metal cation (Fe^2+^, Ni^2+^, Cu^2+^, Cu^+^, and Zn^2+^). The optimizations yield a unique coordination mode (O_3_, O_2_) independent of the nature of the ligand. In the whole, the optimization leads to longer M-O_2_ bond distances, compared to those of M-O_3_ bonds in gas-phase. Our results indicate that the M^2+^-O_i_ (*i* = 2,3) bond distances yielded by B3LYP/ Mixed II are shorter than those relative to B3LYP/Mixed I due to the poor description of the electron-electron repulsion by this latter. The variations of the B3LYP/Mixed I geometrical parameters are very variable in solution-phase. The metal chelation slightly induces an increase of the X-H bond distances that leads to the enhancement of the antioxidant activities of ligands. This shortening of the X-H bond is in agreement with the major drop of the PA values observed when passing from ligands to complexes in gas-phase. The highly negative values of the complexation energies of the M-ADPHT complexes in various media showed that the metal chelation is exothermic. The authors' data revealed that the solvation of complexes drops these complexation energies. This diminution is more pronounced for protic solvents. The highest affinity is obtained for Ni^2+^. From our calculations, we conclude that the formation of M^n+^-ADPHT complexes (*n* = 1, 2) is spontaneous. The HOMO-LUMO gap values reveal that deprotonated ADPHT ligand-M^2+^-complexes are the most stable ones in gas-phase. The highest value of the HOMO-LUMO gap is obtained for the monodental Cu^2+^ complexes obtained. The variation of the values of this gap revealed the fact that the solvation enhances the stability of the complexes. This enhancement is more pronounced in protic solvents. For the neutral ligands, the metal chelation does not affect the increasing order of the HOMO values (with exception made to Ni^2+^) in gas-phase. Our results also show that the solvation of complexes augments the HOMO values and therefore enhances the antioxidant activity of the complexes. The calculated NBO charge on metal (II) atom illustrates a clear relationship between greater electron transfer from the electron donor to the acceptor and higher stability. The metal cation then plays an oxidation role towards both ligands. The MIA value varies proportionately with the retained charge of the metal ion for deprotonated ADPHT ligands. The comparison of calculated energies of hyperconjugative interaction for neutral ADPHT ligands and those of its complexes exhibits the noteworthy influence of the metal chelation on the interaction within both ligands used. The topological parameters yielded from Quantum Theory of Atom in Molecules (QTAIM) indicate that metal-ligand bonds are partly covalent.

## Supplementary Material

Figure 1S reveals the fact that the neutral ligands adopted only the coordination (O2, O3). In the whole, Table 1S-3S display the fact that the impact of the substitution on the geometrical parameters of deprotonated ADPHT ligand-metal complexes is almost minor for divalent metal cation. The highest M-O2 bond distance obtained for 8A complex is due to the monodental preference of Cu+ cation. Higher X-H bond distances observed (Figure 2S) for C1-H1 revealed that these bonds are more labile in gas phase. This fact is not affected by the solvation (Figure 3S). The analysis of LUMO-HOMO gap values (Table 4S) reveals that the neutral ligands are more stable than their deprotonated homologues. The NBO analysis exhibits the fact that the metal chelation highly reduces the interaction between atoms around the metal cation and the adjacent bonds (Table 5S). In the same vein, it is important to underlined that this chelation also decreases the proton affinity and proton affinity free energy. This indicates the enhancement of the antioxidant capacity by this chelation.

## Figures and Tables

**Table 1:** Selected B3LYP/Mixed I level: bond lengths (Ǻ), bond angles (degree), and dihedral angles (degree) for neutral ADPHT ligand-metal complexes in various media.

	Fe^2+^			Ni^2+^			Cu^2+^			Cu^+^			Zn^2+^		
Parameters	1a	1b	1c	2a	2b	2c	3a	3b	3c	4a	4b	4c	5a	5b	5c

*gas*															
M^2+^-O_2_	1.840	1.836	1.835	1.837	1.836	1.835	2.061	2.046	2.040	1.998	1.999	1.999	1.906	1.906	1.905
M^2+^-O_3_	1.818	1.810	1.806	1.827	1.820	1.819	1.969	1.969	1.969	1.995	1.986	1.986	1.899	1.898	1.899
O_2_-M^2+^-O_3_	105.4	105.8	105.8	102.6	102.1	102.1	98.5	98.5	98.3	105.6	106.2	105.6	106.1	106.1	106.4
N_1_-C_3_-O_3_-M^2+^	27.2	24.5	23.4	54.4	−57.3	57.9	57.4	59.1	59.0	51.1	51.6	53.4	42.7	44.9	45.5
N_3_-C_2_-O_2_-M^2+^	33.7	31.7	31.0	35.0	−35.6	35.5	53.1	53.3	52.1	53.3	53.5	54.0	44.9	42.3	42.4
N_1_-N_3_-C_2_-O_2_	12.6	12.3	12.1	11.9	−11.5	11.7	5.7	5.0	6.1	7.7	7.5	7.1	9.8	10.0	9.8
*Water*															
M^2+^-O_2_	1.920	1.925	1.933	1.854	1.863	1.865	1.954	1.951	1.955	2.029	2.035	2.038	2.048	2.047	2.071
M^2+^-O_3_	1.929	1.916	1.932	1.838	1.829	1.835	1.933	1.927	1.928	2.022	2.016	2.019	2.032	2.055	2.023
O_2_-M^2+^-O_3_	99.7	98.2	97.8	97.9	98.3	98.2	101.0	99.7	99.8	103.5	102.9	102.3	90.7	91.1	91.3
N_1_-C_3_-O_3_-M^2+^	44.4	40.9	48.5	64.3	−66.4	66.7	58.6	60.8	63.0	54.5	56.9	59.8	64.3	57.6	66.5
N_3_-C_2_-O_2_-M^2+^	47.5	50.9	54.1	22.8	−36.0	37.2	43.8	44.6	47.6	60.6	61.5	61.8	63.2	66.1	62.0
N_1_-N_3_-C_2_-O_2_	14.1	14.4	11.4	13.4	52.0	10.1	13.6	14.6	11.4	5.7	4.2	3.2	1.0	5.0	1.0
*Benzene*															
M^2+^-O_2_	1.865	1.868	1.871	1.833	1.843	1.841	2.042	2.019	2.018	2.012	2.018	2.018	—	1.944	1.949
M^2+^-O_3_	1.850	1.846	1.846	1.829	1.818	1.820	1.971	1.972	1.970	2.007	2.001	2.001	—	1.928	1.932
O_2_-M^2+^-O_3_	102.6	102.8	103.3	99.6	100.9	100.9	98.9	99.7	98.5	104.5	103.5	103.2	—	101.9	102.3
N_1_-C_3_-O_3_-M^2+^	131.8	32.9	35.4	59.9	−58.5	59.8	58.3	59.2	61.3	52.7	55.9	57.5	—	42.5	48.4
N_3_-C_2_-O_2_-M^2+^	36.1	40.3	38.5	27.9	−31.8	34.7	53.2	50.6	54.7	55.4	58.6	58.6	—	40.8	42.9
N_1_-N_3_-C_2_-O_2_	13.9	14.6	13.1	9.3	−14.0	13.6	5.8	7.2	5.2	9.8	4.6	4.7	—	13.6	9.7
*DMF*															
M^2+^-O_2_	1.918	1.923	1.909	1.852	1.861	1.857	1.958	1.955	1.958	2.028	2.034	2.037	2.043	2.050	2.058
M^2+^-O_3_	1.926	1.912	1.902	1.837	1.828	1.828	1.937	1.930	1.931	2.021	2.015	2.019	2.027	2.042	2.003
O_2_-M^2+^-O_3_	99.6	116.8	100.2	98.1	98.4	98.4	101.0	100.9	99.8	103.6	103.0	102.2	90.9	91.0	91.4
N_1_-C_3_-O_3_-M^2+^	42.4	50.0	38.4	63.9	−66.2	65.7	58.3	60.5	62.9	54.4	56.9	59.8	64.0	57.6	62.8
N_3_-C_2_-O_2_-M^2+^	48.1	40.4	39.9	23.5	−35.6	34.8	44.4	44.8	47.7	60.5	61.0	61.6	63.1	65.8	64.2
N_1_-N_3_-C_2_-O_2_	13.6	14.6	16.6	13.5	−9.5	9.7	13.3	14.5	11.3	5.6	4.5	3.3	1.0	5.0	3.0

**Table 2:** Values (in kJ/mol) of metal binding energy (*D*_*e*_), metal binding enthalpy (Δ*H*_298_^0^), and metal binding free energy (Δ*G*_298_^0^) for ADPHT ligand-metal complexes in various media used at B3LYP/Mixed I level.

		Fe^2+^		Ni^2+^		Cu^2+^		Cu^+^		Zn^2+^	
		**1a**	**6A**	**2a**	**7A**	**3a**	**8A**	**4a**	**9A**	**5a**	**10A**

*D*_*e*_	Gas	−1194	−2081	−1282	−2158	−1215	−2112	−309	−756	−904	−1752
	Water	−277	−435	−267	−443	−421	−642	−121	−211	−57	−177
	Benzene	−642	−1095	−681	−1172	−757	−1284	−201	−451	—	−819
	DMF	−287	−484	−280	−467	−432	−664	−124	−219	−62	−196
Δ*H*_298_^0^	Gas	−1196	−2083	−1285	−2160	−1218	−2114	−312	−759	−907	−1754
	Water	−279	−438	−270	−446	−424	−645	−124	−214	−59	−179
	benzene	−644	−1097	−683	−1174	−759	−1287	−204	−453	—	−821
	DMF	−289	−486	−282	−469	434	−666	−126	−222	−68	−198
Δ*G*_298_^0^	Gas	−1152	−2043	−1182	−2121	−274	−2072	−1242	−722	−864	−1714
	Water	−236	−396	−384	−405	−88	−605	−227	−180	−13	−140
	benzene	−598	−1058	−719	−1132	−165	−1245	−634	−416	—	−778
	DMF	−246	−446	−395	−428	−91	−629	−240	−187	−21	−159

		**1b**	**6B**	**2b**	**7B**	**3b**	**8B**	**4b**	**9B**	**5b**	**10B**

*D*_*e*_	Gas	−1219	−2093	−1304	−2169	−1231	−2063	−318	−758	−926	−1763
	Water	−274	−383	−269	−448	−422	−606	−119	−211	−56	−825
	Benzene	−653	−1138	−691	−1180	−764	−1241	−204	−452	−382	—
	DMF	−285	−459	−282	−471	−433	−628	−122	−219	−64	—
Δ*H*_298_^0^	Gas	−1222	−2095	−1307	−2171	−1233	−2066	−320	−760	−929	−1765
	Water	−277	−385	−272	−450	−425	−609	−121	−213	−58	—
	Benzene	−656	−1141	−694	−1183	−767	−1243	−207	−455	−385	−828
	DMF	−288	−461	−284	−474	−435	−630	−124	−222	−67	—
Δ*G*_298_^0^	Gas	−1177	−2053	−1199	−2130	−283	−2029	−1265	−721	−887	−1726
	Water	−233	−344	−382	−409	−85	−578	−228	−179	−15	—
	Benzene	−608	−1103	−728	−1144	−171	−1211	−647	−421	−334	−789
	DMF	−244	−414	−392	−427	−88	−595	−240	−182	−24	—

		**1c**	**6C**	**2c**	**7C**	**3c**	**8C**	**4c**	**9C**	**5c**	**10C**

*D*_*e*_	Gas	−1226	−2093	−1310	−2168	−1234	−2061	−317	−755	−933	−1764
	Water	−274	—	−264	−444	−417	−602	−114	−207	−53	−175
	Benzene	−655	−1140	−692	−1179	−761	−1239	−202	−451	−383	−825
	DMF	−282	−485	−281	−467	−427	−6024	−117	−215	−62	−194
Δ*H*_298_^0^	Gas	−1229	2096	−1312	−2170	−1237	−2063	−319	−757	−936	−1766
	Water	−277	—	−266	−446	−420	−604	−117	−210	−55	−177
	Benzene	−658	−1142	−695	−1181	−764	−1241	−204	−453	−385	−827
	DMF	−284	−487	−267	−470	−430	−629	−119	−218	−65	−197
Δ*G*_298_^0^	Gas	−1182	−2055	−1201	−2129	−280	−2027	−1268	−718	−893	−1728
	Water	−231	—	−376	−404	−76	−573	−219	−173	−14	−137
	Benzene	−608	−1103	−722	−1142	−163	−1209	−642	−416	−335	−790
	DMF	−240	−446	−385	−428	−79	−594	−239	−181	−22	−158

**Table 3:** NBO charge (q/e) carried by the metal ion, the orbital frontier eigenvalues (eV), LUMO-HOMO gap (Δ*E* in eV), and dipole moments (Debye) for ADPHT ligand-metal complexes in various media at B3LYP/Mixed I level.

	Q/e	*E*_HOMO_ (eV)	*E*_LUMO_ (eV)	Δ*E* (eV)	*µ* (Debye)		Q/e	*E*_HOMO_ (eV)	*E*_LUMO_ (eV)	Δ*E* (eV)	*µ* (Debye)
*Gas phase*											
**1a**	1.23	−13.08	−12.42	0.66	6.50	**6A**	1.06	−9.23	−8.04	1.19	8.09
**1b**	1.22	−13.02	−12.27	0.75	6.41	**6B**	0.81	−9.25	−7.89	1.36	7.46
**1c**	1.22	−12.96	−12.19	0.77	6.95	**6C**	1.05	−9.23	−7.85	1.38	7.60
**2a**	1.05	−13.60	−12.70	0.90	1.79	**7A**	0.95	−9.63	−8.50	1.13	4.63
**2b**	1.05	−13.49	−12.59	0.99	1.89	**7B**	0.95	−9.58	−8.41	1.17	4.51
**2c**	1.04	−13.49	−12.50	0.99	2.52	**7C**	0.95	−9.57	−8.40	1.17	4.92
**3a**	0.92	−13.76	−12.85	0.91	7.87	**8A**	0.75	−10.65	−8.16	2.49	2.27
**3b**	0.92	−13.63	−12.70	0.93	7.09	**8B**	0.85	−9.78	−8.41	1.37	2.71
**3c**	0.92	−13.59	−12.63	0.96	6.93	**8C**	0.85	−9.75	−8.38	1.37	2.98
**5a**	1.61	−12.86	−12.17	0.69	7.92	**10A**	1.50	−9.05	−8.15	0.9	9.57
**5b**	1.62	−12.80	−12.04	0.76	7.98	**10B**	1.49	−9.05	−8.03	1.02	9.05
**5c**	1.61	−12.75	−11.98	0.77	8.58	**10C**	1.49	−9.03	−7.99	1.04	9.27
*Water*											
**1a**	1.58	−7.21	−4.33	2.88	15.03	**6A**	1.41	−6.68	−3.39	3.29	15.17
**1b**	1.57	−7.20	−4.28	2.92	14.85	**6B**	1.37	−6.24	−3.52	2.72	12.45
**1c**	1.58	−7.21	−4.31	2.90	15.98	**6C**	0.90	−7.21	−4.31	2.9	15.98
**2a**	1.36	−7.28	−5.40	1.88	1.36	**7A**	1.41	−6.74	−4.47	2.27	12.93
**2b**	1.35	−7.29	−5.37	1.92	1.35	**7B**	1.37	−6.73	−4.42	2.31	12.14
**2c**	1.35	−7.30	−5.38	1.92	1.35	**7C**		−6.74	−4.41	2.33	12.93
**3a**	1.22	−7.67	−6.49	1.18	4.92	**8A**	1.25	−7.18	−4.73	2.45	3.64
**3b**	1.24	−7.48	−6.46	1.02	7.11	**8B**	1.25	−6.85	−−5.04	1.81	7.60
**3c**	1.24	−7.48	−6.46	1.02	7.79	**8C**	1.25	−6.84	−5.03	1.81	7.80
**5a**	1.61	−7.09	−2.22	4.87	17.97	**10A**	1.79	−6.50	−1.76	4.74	18.17
**5b**	1.62	−7.11	−2.30	4.81	17.56	**10B**					
**5c**	1.61	−7.11	−2.29	4.82	18.40	**10C**	1.78	6.48	1.75	4.73	18.3
*Benzene*											
**1a**	1.39	−9.58	−8.21	1.37	10.68	**6A**	1.10	−7.71	−5.63	2.08	10.03
**1b**	1.49	−9.59	−8.10	1.49	10.24	**6B**		−7.70	−5.64	2.06	10.7
**1c**	1.51	−9.60	−8.09	1.51	10.95	**6C**	1.22	−7.66	−5.62	2.04	11.00
**2a**	1.19	−9.90	−8.82	1.08	6.90	**7A**	1.09	7.84	6.45	1.39	8.57
**2b**	1.18	−9.87	−8.75	1.12	6.70	**7B**		−7.82	−6.37	2.04	8.29
**2c**	1.18	−9.83	−8.73	1.10	7.45	**7C**	1.08	−7.82	−6.36	1.46	8.77
**3a**	0.97	−10.37	−9.23	1.14	6.43	**8A**	0.38	−8.73	−6.24	2.49	2.67
**3b**	0.99	−10.32	−9.18	1.14	5.28	**8B**	0.42	−7.97	−6.52	1.45	5.51
**3c**	0.99	−10.29	−9.16	1.13	5.74	**8C**	0.88	−7.97	−6.51	1.46	5.65
**5a**						**10A**	1.65	−7.49	−5.04	2.45	13.39
**5b**	1.76	−9.44	−6.93	2.51	12.3	**10B**	1.64	−7.50	−4.97	2.53	12.79
**5c**	1.75	−9.44	−6.97	2.47	13.4	**10C**	1.76	−7.50	−5.00	2.50	13.21
*DMF*											
**1a**	1.57	−7.28	−4.48	2.80	14.83	**6A**	—	—	—	—	—
**1b**	1.56	−7.28	−4.42	2.86	14.65	**6B**	1.40	−6.68	−3.45	3.23	14.10
**1c**	1.55	−7.28	−4.39	2.89	16.04	**6C**	1.47	−6.58	−3.37	3.21	15.57
**2a**	1.35	−7.36	−5.54	1.82	12.21	**7A**	1.24	−6.77	−4.55	2.22	12.78
**2b**	1.34	−7.37	−5.50	1.87	12.02	**7B**	1.24	−6.76	−4.50	2.26	11.99
**2c**	1.18	−7.37	−5.48	1.89	12.66	**7C**	1.24	−6.77	−4.49	2.28	12.75
**3a**	1.20	−7.81	−6.58	1.23	4.08	**8A**	0.80	−7.23	−4.77	2.46	3.60
**3b**	1.22	−7.61	−6.55	1.06	6.28	**8B**	0.93	−6.88	−5.08	1.8	7.54
**3c**	1.22	−7.59	−6.55	1.04	6.70	**8C**	0.92	−6.88	−5.08	1.8	7.73
**5a**	1.85	−7.16	−2.46	4.70	17.77	**10A**	1.79	−6.52	−1.91	4.61	18.18
**5b**	1.85	−7.18	−2.47	4.71	17.37	**10B**	1.85	−7.18	−2.47	4.71	17.37
**5c**	1.85	−7.18	−2.45	4.73	18.23	**10C**	1.78	6.52	1.88	4.64	18.11

**Table 4:** Topological parameters of the metal-ligand for optimized structures at B3LYP/Mixed II level for neutral ADPHT-M^2+^-complexes.

	M^2+^-O_2_					M^2+^-O_3_				
	**1a**	**2a**	**3a**	**4a**	**5a**	**1a**	**2a**	**3a**	**4a**	**5a**

*ρ*(*r*)	0.121	0.133	0.108	0.107	0.112	0.134	0.133	0.113	0.106	0.113
∇^2^(*ρ*(*r*))	0.852	0.902	0.564	0.550	0.510	0.852	0.878	0.589	0.526	0.510
*H*(*r*)	−0.009	−0.022	−0.036	−0.038	−0.047	−0.018	−0.022	−0.037	−0.039	−0.048
*G*(*r*)	0.222	0.247	0.176	0.175	0.175	0.230	0.247	0.176	0.170	0.176
*ν*(*r*)	−0.231	−0.269	−0.212	−0.213	−0.222	−0.248	−0.269	−0.212	−0.209	−0.224
−*G*(*r*)/*ν*(*r*)	0.962	0.920	0.833	0.823	0.787	0.929	0.920	0.833	0.815	0.785

	**1b**	**2b**	**3b**	**4b**	**5b**	**1b**	**2b**	**3b**	**4b**	**5b**

*ρ*(*r*)	0.124	0.133	0.109	0.108	0.113	0.133	0.135	0.115	0.106	0.115
∇^2^(*ρ*(*r*))	0.846	0.908	0.572	0.552	0.516	0.856	0.888	0.600	0.527	0.521
*H*(*r*)	−0.011	−0.022	−0.036	−0.038	−0.048	−0.017	−0.023	−0.037	−0.039	−0.049
*G*(*r*)	0.223	0.249	0.179	0.176	0.177	0.231	0.245	0.187	0.170	0.229
*ν*(*r*)	−0.234	−0.270	−0.214	−0.213	−0.225	−0.248	−0.269	−0.225	−0.209	−0.229
−*G*(*r*)/*ν*(*r*)	0.953	0.920	0.834	0.823	0.787	0.931	0.913	0.834	0.815	0.785

	**1c**	**2c**	**3c**	**4c**	**5c**	**1c**	**2c**	**3c**	**4c**	**5c**

*ρ*(*r*)	0.122	1.34	0.110	0.108	0.113	0.137	0.136	0.116	0.107	0.115
∇^2^(*ρ*(*r*))	0.856	0.911	0.576	0.552	0.517	0.877	0.890	0.602	0.528	0.522
*H*(*r*)	−0.009	−0.022	−0.036	−0.038	−0.048	−0.019	−0.024	−0.038	−0.039	−0.049
*G*(*r*)	0.223	0.250	0.180	0.176	0.177	0.238	0.246	0.188	0.171	0.180
*ν*(*r*)	−0.233	−0.271	−0.215	−0.213	−0.225	−0.257	−0.270	−0.226	−0.209	−0.230
−*G*(*r*)/*ν*(*r*)	0.959	0.920	0.834	0.823	0.787	0.926	0.912	0.834	0.815	0.785

**Table 5:** Topological parameters of the metal-ligand for optimized structures at B3LYP/Mixed II level for deprotonated ADPHT-M^2+^-complexes.

	M^2+^-O_2_				M^2+^-O_3_			
	**6A**	**7A**	**8A**	**9A**	**6A**	**7A**	**8A**	**9A**

*ρ*(*r*)	0.162	0.158	0.138	0.132	0.131	0.139	0.125	0.120
∇^2^(*ρ*(*r*))	1.019	1.004	0.759	0.634	0.865	0.935	0.679	0.552
*H*(*r*)	−0.035	−0.036	−0.045	−0.058	−0.015	−0.024	−0.039	−0.052
*G*(*r*)	0.290	0.287	0.235	0.216	0.232	0.258	0.210	0.190
*ν*(*r*)	−0.325	−0.322	−0.281	−0.274	−0.247	−0.282	−0.248	−0.242
−*G*(*r*)/*ν*(*r*)	0.893	0.889	0.838	0.790	0.937	0.914	0.842	0.784

	**6B**	**7B**	**8B**	**9B**	**6B**	**7B**	**8B**	**9B**

*ρ*(*r*)	0.162	0.158	0.138	0.132	0.133	0.140	0.126	0.121
∇^2^(*ρ*(*r*))	1.017	1.003	0.762	0.633	0.869	0.932	0.684	0.556
*H*(*r*)	−0.035	−0.036	−0.046	−0.058	−0.016	−0.025	−0.040	−0.053
*G*(*r*)	0.289	0.286	0.236	0.216	0.234	0.258	0.211	0.191
*ν*(*r*)	−0.324	−0.322	−0.282	−0.274	−0.250	−0.283	−0.251	−0.244
−*G*(*r*)/*ν*(*r*)	0.892	0.889	0.838	0.789	0.934	0.911	0.841	0.784

	**6C**	**7C**	**8C**	**9C**	**6C**	**7C**	**8C**	**9C**

*ρ*(*r*)	0.162	0.158	0.138	0.132	0.133	0.141	0.126	0.121
∇^2^(*ρ*(*r*))	1.017	1.005	0.758	0.634	0.870	0.933	0682	0.554
*H*(*r*)	−0.035	−0.036	−0.046	−0.058	−0.017	−0.025	−0.040	−0.053
*G*(*r*)	0.289	0.287	0.235	0.216	0.234	0.259	0.210	0.191
*ν*(*r*)	−0.324	−0.323	−0.281	−0.274	−0.251	−0.284	−0.250	−0.244
−*G*(*r*)/*ν*(*r*)	0.892	0.889	0.837	0.789	0.933	0.911	0.840	0.784
