# Supplementary material for: A DFT Study of Structural and Bonding Properties of Complexes Obtained from First-Row Transition Metal Chelation by 3-Alkyl-4-phenylacetylamino-4,5-dihydro-1H-1,2,4-triazol-5-one and Its Derivatives
Source: Bioinorg Chem Appl. 2017 Jul 3;2017:5237865. doi: 10.1155/2017/5237865 (PMC5512031; doi:10.1155/2017/5237865)
Supplement: Supplementary file 1 — Figure 1S reveals the fact that the neutral ligands adopted only the coordination (O2, O3). In the whole, Table 1S-3S display the fact that the impact of the substitution on the geometrical parameters of deprotonated ADPHT ligand-metal complexes is almost minor for divalent metal cation. The highest M-O2 bond distance obtained for 8A complex is due to the monodental preference of Cu+ cation. Higher X-H bond distances observed (Figure 2S) for C1-H1 revealed that these bonds are more labile in gas phase. This fact is not affected by the solvation (Figure 3S). The analysis of LUMO-HOMO gap values (Table 4S) reveals that the neutral ligands are more stable than their deprotonated homologues. The NBO analysis exhibits the fact that the metal chelation highly reduces the interaction between atoms around the metal cation and the adjacent bonds (Table 5S). In the same vein, it is important to underlined that this chelation also decreases the proton affinity and proton affinity free energy. This indicates the enhancement of the antioxidant capacity by this chelation. [file 5237865.f1.docx]

**SUPPORTING INFORMATION**

**Table S1**: Selected B3LYP/Mixed I bond lengths (Ǻ) for depronated ADPHT ligand- metal complexes.

**Table S2** : Selected B3LYP/Mixed I bond angles (degrees) for depronated ADPHT ligand- metal complexes.

**Table S3** : Selected B3LYP/Mixed I dihedral angles (degrees) for depronated ADPHT ligand- metal complexes.

**Table S4**: Orbital frontier eigenvalues (eV), LUMO-HOMO gap (ΔE in eV) and dipole moments (Debye) for ADPHT ligands in various media using B3LYP/Mixed I.

**Table S5** : B3LYP/Mixed I calculated energy of hyperconjugative interaction (in kJ/mol) for ADPHT ligands and its complexes by NBO analysis.

**Table S6**: Solvation enthalpies of proton H^+^ ($\Delta_{f}H_{Sol}^{o}(H^{+})$in kJ/mol) and solvation free energy of proton H^+^ ($\Delta_{f}G_{Sol}^{o}(H^{+})$in kJ/mol).

**Fig.1S**: Optimized structures for neutral ADPHT ligand- metal complexes at B3LYP/Mixed I level.

**Fig. 2S:** X-H (X = N_2_, N_3_ and C_1_) bond distances for ADPHT ligand- M-ADPHT complexes at B3LYP/Mixed I

**Fig. 3S:** C_1_-H_1_ bond distance in different media at B3LYP/Mixed I

**Table S1:** Selected B3LYP/ Mixed I bond lengths (Ǻ) for depronated ADPHT ligand- metal complexes.

|  | Fe^2+^ | | | Ni^2+^ | | | Cu^2+^ | | | Cu^+^ | | | Zn^2+^ | | |
| --- | --- | --- | --- | --- | --- | --- | --- | --- | --- | --- | --- | --- | --- | --- | --- |
| Parameters | **6A** | **6B** | **6C** | **7A** | **7B** | **7C** | **8A** | **8B** | **8C** | **9A** | **9B** | **9C** | **10A** | **10B** | **10C** |
| Gas |  |  |  |  |  |  |  |  |  |  |  |  |  |  |  |
| M-O_2_ | 1,750 | 1,750 | 1,750 | 1,766 | 1,766 | 1,766 | 2,657 | 1,947 | 1,948 | 1,903 | 1,904 | 1,905 | 1,850 | 1,850 | 1,850 |
| M-O_3_ | 1,812 | 1,807 | 1,806 | 1,806 | 1,802 | 1,800 | 1,944 | 1,973 | 1,972 | 1,952 | 1,950 | 1,949 | 1,899 | 1,895 | 1,895 |
| O_2_-C_2_ | 1,331 | 1,331 | 1,332 | 1,310 | 1,313 | 1,313 | 1,225 | 1,256 | 1,255 | 1,293 | 1,294 | 1,293 | 1,333 | 1,334 | 1,334 |
| O_3_-C_3_ | 1,283 | 1,285 | 1,286 | 1,281 | 1,284 | 1,285 | 1,239 | 1,243 | 1,243 | 1,266 | 1,268 | 1,268 | 1,292 | 1,294 | 1,294 |
| C_4_-N_1_ | 1,393 | 1,408 | 1,410 | 1,387 | 1,399 | 1,402 | 1,396 | 1,413 | 1,415 | 1,377 | 1,388 | 1,390 | 1,386 | 1,400 | 1,401 |
| C_4_-N_4_ | 1,296 | 1,301 | 1,302 | 1,299 | 1,305 | 1,306 | 1,292 | 1,296 | 1,297 | 1,302 | 1,306 | 1,307 | 1,300 | 1,305 | 1,306 |
| N_3_-C_2_ | 1,292 | 1,292 | 1,291 | 1,297 | 1,297 | 1,297 | 1,403 | 1,365 | 1,366 | 1,315 | 1,315 | 1,315 | 1,296 | 1,296 | 1,296 |
| Benzene |  |  |  |  |  |  |  |  |  |  |  |  |  |  |  |
| M-O_2_ | 1,754 | 1,786 | 1,784 | 1,769 | 1,770 | 1,773 | 2,864 | 1,966 | 1,968 | 1,920 | 1,920 | 1,922 | 1,883 | 1,885 | 1,884 |
| M-O_3_ | 1,803 | 1,835 | 1,840 | 1,804 | 1,807 | 1,807 | 1,935 | 1,995 | 1,995 | 1,968 | 1,965 | 1,967 | 1,931 | 1,921 | 1,932 |
| O_2_-C_2_ | 1,323 | 1,322 | 1,323 | 1,318 | 1,317 | 1,315 | 1,223 | 1,251 | 1,250 | 1,292 | 1,293 | 1,293 | 1,318 | 1,319 | 1,320 |
| O_3_-C_3_ | 1,281 | 1,281 | 1,281 | 1,281 | 1,284 | 1,285 | 1,239 | 1,240 | 1,240 | 1,265 | 1,267 | 1,267 | 1,281 | 1,285 | 1,287 |
| C_4_-N_1_ | 1,390 | 1,403 | 1,404 | 1,385 | 1,395 | 1,396 | 1,394 | 1,411 | 1,413 | 1,376 | 1,386 | 1,387 | 1,384 | 1,396 | 1,395 |
| C_4_-N_4_ | 1,298 | 1,302 | 1,302 | 1,299 | 1,307 | 1,307 | 1,292 | 1,296 | 1,297 | 1,303 | 1,307 | 1,308 | 1,300 | 1,305 | 1,306 |
| N_3_-C_2_ | 1,294 | 1,292 | 1,292 | 1,296 | 1,296 | 1,297 | 1,406 | 1,372 | 1,373 | 1,315 | 1,315 | 1,315 | 1,299 | 1,299 | 1,299 |
| DMF |  |  |  |  |  |  |  |  |  |  |  |  |  |  |  |
| M-O_2_ | 1,871 | 1,836 | 1,871 | 1,792 | 1,792 | 1,792 | 3,144 | 1,974 | 1,977 | 1,944 | 1,944 | 1,946 | 1,957 | * * | 1,948 |
| M-O_3_ | 1,902 | 1,896 | 1,892 | 1,825 | 1,822 | 1,823 | 1,938 | 2,009 | 2,012 | 1,994 | 1,989 | 1,993 | 2,002 |  | 2,005 |
| O_2_-C_2_ | 1,305 | 1,317 | 1,307 | 1,312 | 1,317 | 1,314 | 1,222 | 1,250 | 1,249 | 1,290 | 1,291 | 1,290 | 1,296 |  | 1,298 |
| O_3_-C_3_ | 1,272 | 1,271 | 1,274 | 1,278 | 1,279 | 1,280 | 1,238 | 1,238 | 1,238 | 1,263 | 1,265 | 1,264 | 1,266 |  | 1,271 |
| C_4_-N_1_ | 1,378 | 1,390 | 1,390 | 1,379 | 1,390 | 1,391 | 1,392 | 1,407 | 1,408 | 1,374 | 1,383 | 1,385 | 1,375 |  | 1,386 |
| C_4_-N_4_ | 1,302 | 1,306 | 1,306 | 1,303 | 1,307 | 1,308 | 1,293 | 1,297 | 1,298 | 1,304 | 1,308 | 1,309 | 1,304 |  | 1,308 |
| N_3_-C_2_ | 1,304 | 1,297 | 1,302 | 1,299 | 1,298 | 1,298 | 1,409 | 1,370 | 1,371 | 1,317 | 1,317 | 1,318 | 1,309 |  | 1,308 |
| water |  |  |  |  |  |  |  |  |  |  |  |  |  |  |  |
| M-O_2_ | 1,841 | 1,801 | ** | 1,793 | 1,794 | 1,794 | 3,143 | 1,973 | 1,977 | 1,946 | 1,945 | 1,947 | 1,957 | ** | 1,950 |
| M-O_3_ | 1,902 | 1,843 |  | 1,826 | 1,823 | 1,824 | 1,938 | 2,008 | 2,012 | 1,996 | 1,991 | 1,994 | 1,996 |  | 2,009 |
| O_2_-C_2_ | 1,314 | 1,315 |  | 1,312 | 1,317 | 1,313 | 1,222 | 1,250 | 1,249 | 1,289 | 1,291 | 1,290 | 1,296 |  | 1,297 |
| O_3_-C_3_ | 1,273 | 1,279 |  | 1,278 | 1,279 | 1,280 | 1,238 | 1,238 | 1,238 | 1,263 | 1,264 | 1,264 | 1,265 |  | 1,270 |
| C_4_-N_1_ | 1,379 | 1,393 |  | 1,378 | 1,390 | 1,391 | 1,392 | 1,406 | 1,408 | 1,374 | 1,383 | 1,384 | 1,375 |  | 1,386 |
| C_4_-N_4_ | 1,302 | 1,306 |  | 1,303 | 1,307 | 1,308 | 1,293 | 1,297 | 1,298 | 1,304 | 1,308 | 1,309 | 1,303 |  | 1,308 |
| N_3_-C_2_ | 1,299 | 1,299 |  | 1,299 | 1,298 | 1,299 | 1,408 | 1,370 | 1,371 | 1,318 | 1,317 | 1,318 | 1,309 |  | 1,309 |

**Table S2** : **:** Selected B3LYP/ Mixed I bond angles (degrees) for depronated ADPHT ligand- metal complexes.

|  | Fe^2+^ | | | Ni^2+^ | | | Cu^2+^ | | | Cu^+^ | | | Zn^2+^ | | |
| --- | --- | --- | --- | --- | --- | --- | --- | --- | --- | --- | --- | --- | --- | --- | --- |
| Parameters | **6A** | **6B** | **6C** | **7A** | **7B** | **7C** | **8A** | **8B** | **8C** | **9A** | **9B** | **9C** | **10A** | **10B** | **10C** |
| Gaz |  |  |  |  |  |  |  |  |  |  |  |  |  |  |  |
| O_2_-M-O_3_ | 111,2 | 111,6 | 111,6 | 108,3 | 108,0 | 107,7 | 77,5 | 103,6 | 103,2 | 120,9 | 121,5 | 120,9 | 113,7 | 114,2 | 114,2 |
| M-O_2_-C_2_ | 133,7 | 133,0 | 132,6 | 129,3 | 128,6 | 128,5 | 85,0 | 128,0 | 128,0 | 111,4 | 110,4 | 111,0 | 119,7 | 118,6 | 118,4 |
| M-O_3_-C_3_ | 124,3 | 124,9 | 124,5 | 121,6 | 121,0 | 120,6 | 136,8 | 122,7 | 122,5 | 106,0 | 105,8 | 105,8 | 113,9 | 113,7 | 113,3 |
| O_2_-C_2_-N_3_ | 129,1 | 129,3 | 129,2 | 129,2 | 128,9 | 129,0 | 121,9 | 127,8 | 127,7 | 131,1 | 131,1 | 131,2 | 128,9 | 128,8 | 128,8 |
| O_3_-C_3_-N_1_ | 131,1 | 131,0 | 130,9 | 129,9 | 129,5 | 129,4 | 130,0 | 130,4 | 130,3 | 131,0 | 130,8 | 130,8 | 130,0 | 129,8 | 129,7 |
| N_1_-C_4_-N_4_ | 112,4 | 110,9 | 110,7 | 112,1 | 110,6 | 110,34 | 1,403 | 111,0 | 110,7 | 113,3 | 112,0 | 111,8 | 112,3 | 110,8 | 110,7 |
| Benzene |  |  |  |  |  |  |  |  |  |  |  |  |  |  |  |
| O_2_-M-O_3_ | 110,0 | 107,4 | 107,3 | 105,8 | 105,4 | 105,4 | 74,4 | 100,5 | 100,5 | 117,5 | 118,4 | 117,1 | 108,1 | 109,2 | 108,0 |
| M-O_2_-C_2_ | 134,1 | 134,1 | 133,7 | 127,5 | 128,7 | 130,5 | 81,2 | 127,3 | 127,4 | 111,7 | 111,0 | 111,2 | 122,9 | 121,8 | 121,0 |
| M-O_3_-C_3_ | 125,3 | 125,6 | 125,2 | 123,1 | 120,5 | 120,2 | 138,4 | 122,4 | 122,0 | 106,6 | 106,4 | 106,8 | 117,6 | 116,8 | 114,5 |
| O_2_-C_2_-N_3_ | 129,5 | 130,4 | 130,3 | 128,6 | 128,6 | 129,3 | 121,8 | 126,0 | 126,1 | 130,5 | 130,6 | 130,5 | 129,4 | 129,5 | 128,6 |
| O_3_-C_3_-N_1_ | 131,1 | 131,2 | 131,2 | 130,1 | 129,1 | 129,1 | 129,9 | 129,6 | 129,5 | 130,4 | 130,3 | 130,3 | 130,7 | 130,3 | 129,6 |
| N_1_-C_4_-N_4_ | 112,4 | 111,1 | 110,9 | 112,0 | 110,6 | 110,4 | 111,7 | 110,8 | 110,5 | 113,1 | 111,9 | 111,6 | 112,5 | 111,1 | 110,9 |
| DMF |  |  |  |  |  |  |  |  |  |  |  |  |  |  |  |
| O_2_-M-O_3_ | 100,8 | 100,6 | 99,7 | 103,4 | 102,4 | 102,7 | 68,1 | 99,8 | 99,2 | 112,0 | 112,9 | 111,3 | 94,4 | * * | 98,0 |
| M-O_2_-C_2_ | 127,2 | 130,4 | 124,6 | 129,0 | 124,9 | 127,7 | 76,4 | 125,6 | 126,1 | 112,8 | 111,3 | 111,7 | 124,4 |  | 122,8 |
| M-O_3_-C_3_ | 120,9 | 119,3 | 122,4 | 120,5 | 122,0 | 121,5 | 138,9 | 121,4 | 121,1 | 107,8 | 107,7 | 108,0 | 120,0 |  | 117,2 |
| O_2_-C_2_-N_3_ | 128,7 | 129,2 | 127,9 | 129,2 | 127,8 | 128,7 | 121,8 | 125,5 | 125,5 | 129,6 | 129,6 | 129,4 | 127,7 |  | 128,0 |
| O_3_-C_3_-N_1_ | 129,7 | 129,0 | 130,1 | 129,0 | 129,3 | 129,2 | 129,8 | 129,1 | 129,0 | 129,7 | 129,6 | 129,6 | 129,4 |  | 129,4 |
| N_1_-C_4_-N_4_ | 112,3 | 111,0 | 111,0 | 112,0 | 110,7 | 110,6 | 111,8 | 110,8 | 110,5 | 112,8 | 111,6 | 111,4 | 112,3 |  | 111,1 |
| water |  |  |  |  |  |  |  |  |  |  |  |  |  |  |  |
| O_2_-M-O_3_ | 99,6 | 105,8 | - | 103,3 | 102,3 | 102,6 | 68,1 | 100,2 | 99,2 | 111,6 | 112,7 | 111,1 | 95,0 | * * | 97,7 |
| M-O_2_-C_2_ | 132,5 | 127,0 | - | 129,0 | 124,9 | 127,7 | 76,3 | 125,4 | 126,0 | 112,7 | 111,3 | 111,7 | 123,5 |  | 123,0 |
| M-O_3_-C_3_ | 117,2 | 121,0 | - | 120,5 | 122,0 | 121,5 | 138,7 | 120,9 | 121,1 | 108,0 | 107,7 | 108,1 | 121,5 |  | 117,3 |
| O_2_-C_2_-N_3_ | 129,2 | 128,7 | - | 129,2 | 127,9 | 128,7 | 121,9 | 125,6 | 125,5 | 129,5 | 129,5 | 129,4 | 127,8 |  | 128,0 |
| O_3_-C_3_-N_1_ | 128,9 | 129,6 | - | 128,9 | 129,3 | 129,2 | 129,8 | 129,1 | 129,0 | 129,7 | 129,6 | 129,6 | 130,0 |  | 129,4 |
| N_1_-C_4_-N_4_ | 112,2 | 110,9 | - | 112,0 | 110,8 | 110,6 | 111,8 | 110,8 | 110,6 | 112,8 | 111,6 | 111,4 | 112,5 |  | 111,1 |

**Table S3** : **:** Selected B3LYP/ Mixed I dihedral angles (degrees) for depronated ADPHT ligand- metal complexes

|  | Fe^2+^ | | | Ni^2+^ | | | Cu^2+^ | | | Cu^+^ | | | Zn^2+^ | | |
| --- | --- | --- | --- | --- | --- | --- | --- | --- | --- | --- | --- | --- | --- | --- | --- |
| Parameters | **6A** | **6B** | **6C** | **7A** | **7B** | **7C** | **8A** | **8B** | **8C** | **9A** | **9B** | **9C** | **10A** | **10B** | **10C** |
| Gas |  |  |  |  |  |  |  |  |  |  |  |  |  |  |  |
| M-O_2_-C_2_-N_3_ | 19,7 | 17,8 | 20,5 | 41,6 | -42,8 | 43,2 | -100,0 | 52,1 | 52,6 | 47,7 | 47,7 | 47,3 | 48,8 | 49,2 | 49,6 |
| M-O_3_-C_3_-N_1_ | 16,2 | 14,3 | 16,8 | 36,6 | -40,0 | 42,5 | -9,0 | 36,0 | 38,2 | 38,9 | 38,9 | 40,7 | 39,4 | 39,6 | 40,5 |
| O_3_-C_3_-N_1_-N_3_ | 8,3 | 7,9 | 8,7 | 6,3 | -6,7 | 5,3 | 0,2 | 9,9 | 8,6 | 16,8 | 18,0 | 15,9 | 11,6 | 12,4 | 12,7 |
| N_1_-N_3_-C_2_-O_2_ | 0,3 | 0,4 | 0,7 | 5,7 | -5,9 | 6,2 | 28,4 | -16,9 | -17,1 | 5,2 | 5,6 | 5,8 | 1,3 | 1,7 | 1,5 |
| N_1_-N_3_-C_2_-C_1_ | -179,0 | -179,3 | -179,0 | -173,9 | 173,8 | -173,3 | -151,8 | 171,0 | 171,1 | -174,5 | -174,5 | -174,7 | -176,6 | -176,2 | -176,5 |
| N_3_-C_2_-C_1_-C_5_ | -3,4 | 2,1 | 2,6 | 11,2 | -13,8 | 10,5 | 105,7 | 76,9 | 76,9 | 7,6 | 9,4 | 15,0 | 9,0 | 8,1 | 8,6 |
| Benzene |  |  |  |  |  |  |  |  |  |  |  |  |  |  |  |
| M-O_2_-C_2_-N_3_ | 21,2 | 22,9 | 25,4 | 50,2 | -49,8 | 41,7 | -98,4 | 60,7 | 59,7 | 51,6 | 50,8 | 52,5 | 50,1 | 49,4 | 54,1 |
| M-O_3_-C_3_-N_1_ | 17,0 | 22,5 | 24,1 | 35,3 | -45,6 | 47,5 | -2,2 | 41,8 | 43,5 | 42,5 | 41,9 | 43,8 | 37,7 | 39,5 | 45,3 |
| O_3_-C_3_-N_1_-N_3_ | 4,9 | 10,1 | 10,6 | 6,1 | -7,2 | 5,7 | 0,4 | 10,5 | 9,3 | 15,9 | 17,0 | 14,8 | 10,4 | 12,0 | 13,7 |
| N_1_-N_3_-C_2_-O_2_ | 2,0 | 1,4 | 1,6 | 5,0 | -4,9 | 5,4 | 29,0 | -21,5 | -21,0 | 4,4 | 5,2 | 4,6 | 3,2 | 3,4 | 1,4 |
| N_1_-N_3_-C_2_-C_1_ | -177,4 | -178,6 | -178,3 | -174,0 | 174,6 | -174,2 | -152,2 | 166,9 | 167,5 | -175,0 | -174,9 | -175,7 | -174,2 | 174,6 | -177,7 |
| N_3_-C_2_-C_1_-C_5_ | 3,562 | 2,6 | 2,6 | 10,6 | -13,5 | 15,5 | 100,6 | 80,7 | 79,5 | 5,7 | 11,3 | 13,6 | 2,7 | 3,5 | 16,9 |
| DMF |  |  |  |  |  |  |  |  |  |  |  |  |  |  |  |
| M-O_2_-C_2_-N_3_ | 56,4 | 49,3 | 62,2 | 50,0 | -60,0 | 53,8 | -96,3 | 64,7 | 64,2 | 57,5 | 58,1 | 59,8 | 69,9 | - | 66,2 |
| M-O_3_-C_3_-N_1_ | 46,0 | 50,3 | 42,6 | 48,4 | -44,5 | 47,2 | 16,0 | 44,8 | 47,0 | 47,9 | 46,9 | 49,2 | 54,0 | - | 52,7 |
| O_3_-C_3_-N_1_-N_3_ | 9,5 | 13,8 | 9,0 | 7,5 | -7,8 | 6,0 | -0,6 | 11,3 | 9,8 | 14,0 | 14,8 | 12,7 | 7,3 | - | 10,5 |
| N_1_-N_3_-C_2_-O_2_ | 3,0 | -0,5 | 3,4 | 3,8 | -3,4 | 4,3 | 30,0 | -21,3 | -21,4 | 3,0 | 3,2 | 2,7 | -1,2 | - | -0,1 |
| N_1_-N_3_-C_2_-C_1_ | -175,8 | -178,2 | -175,7 | -174,9 | 174,8 | -174,4 | -152,3 | 167,1 | 167,1 | -176,4 | -176,1 | -176,9 | -179,9 | - | -180,0 |
| N_3_-C_2_-C_1_-C_5_ | 13,7 | -16,2 | 14,0 | 7,9 | -6,2 | 11,8 | 91,2 | 87,4 | 86,9 | 5,0 | 2,8 | 5,4 | 14,8 | - | 19,3 |
| water |  |  |  |  |  |  |  |  |  |  |  |  |  |  |  |
| M-O_2_-C_2_-N_3_ | 45,1 | 50,5 | - | 50,1 | -60,0 | 53,9 | -96,3 | 64,1 | 64,4 | 58,4 | 58,3 | 60,1 | 69,4 | - | 66,3 |
| M-O_3_-C_3_-N_1_ | 53,8 | 40,4 | - | 48,5 | -44,5 | 47,4 | 16,3 | 45,1 | 47,0 | 48,1 | 47,2 | 49,4 | 49,0 | - | 52,9 |
| O_3_-C_3_-N_1_-N_3_ | 13,1 | 10,7 | - | 7,5 | -7,8 | 6,0 | -0,7 | 11,4 | 9,8 | 13,8 | 14,7 | 12,6 | 6,7 | - | 10,5 |
| N_1_-N_3_-C_2_-O_2_ | -1,2 | 2,2 | - | 3,8 | -3,4 | 4,3 | 30,1 | -21,2 | -21,3 | 2,8 | 3,1 | 2,6 | 0,7 | - | -0,1 |
| N_1_-N_3_-C_2_-C_1_ | -179,2 | -176,0 | - | -174,8 | 174,7 | -174,3 | -152,2 | 167,2 | 167,2 | -176,6 | -176,2 | -176,9 | -178,3 | - | -180,0 |
| N_3_-C_2_-C_1_-C_5_ | -4,4 | -6,3 | - | 7,6 | -5,5 | 11,6 | 91,0 | 88,7 | 87,1 | 4,3 | 2,7 | 5,0 | 14,2 | - | 18,7 |

|  | E_HOMO_ | E_LUMO_ | ΔE | µ |  | E_HOMO_ | E_LUMO_ | ΔE | µ |
| --- | --- | --- | --- | --- | --- | --- | --- | --- | --- |
| **gas** |  |  |  |  |  |  |  |  |  |
| **a** | -6.60 | -0.4 | 6.2 | 3.48 | **A** | -6.61 | -4.42 | 2.19 | 3.48 |
| **b** | -6.50 | -0.05 | 6.45 | 3.5 | **B** | -6.43 | -0.05 | 6.38 | 3.51 |
| **c** | -6.45 | -0.04 | 6.41 | 3.7 | **C** | -6.45 | -0.04 | 6.41 | 3.71 |
| **water** |  |  |  |  |  |  |  |  |  |
| **a** | -6.60 | -0.42 | 6.18 | 4.67 | **A** | -5.12 | -0.04 | 5.08 | 11.32 |
| **b** | -6.43 | -2.09 | 4.34 | 4.95 | **B** | -5.08 | -0.04 | 5.04 | 11.65 |
| **c** | -6.42 | -0.22 | 6.20 | 5.14 | **C** | -5.00 | -0.13 | 4.87 | 9.29 |
| **benzene** |  |  |  |  |  |  |  |  |  |
| **a** | -6.61 | -0.42 | 6.19 | 4.00 | **A** | -3.36 | -1.32 | 2.04 | 9.31 |
| **b** | -6.46 | -0.09 | 6.37 | 4.06 | **B** | -3.32 | -1.32 | 2.00 | 9.23 |
| **c** | -6.45 | -0.09 | 6.36 | 4.27 | **C** | -3.21 | -1.49 | 1.72 | 7.20 |
| **DMF** |  |  |  |  |  |  |  |  | 11.24 |
| **a** | -6.60 | -4.20 | 2.4 | 4.63 | **A** | -5.06 | -0.06 | 5.00 | 11.34 |
| **b** | -6.43 | -0.20 | 6.23 | 4.90 | **B** | -5.01 | -0.08 | 4.93 | 11.34 |
| **c** | -6.43 | 0.12 | 6.31 | 5.10 | **C** | -4.93 | -0.18 | 4.75 | 9.19 |

**Table S4:** Orbital frontier eigenvalues (eV), LUMO-HOMO gap (ΔE in eV) and dipole moments (Debye) for ADPHT ligands in various media using B3LYP/ Mixed I.

**Table S5:** B3LYP/ Mixed I calculated energies of hyperconjugative interaction (in kJ/mol) for ADPHT ligands and its complexes by NBO analysis.

| Donor  NBO (i) | Acceptor  NBO(i) | ligands | | | Complexes | | | | | | | | | | | |
| --- | --- | --- | --- | --- | --- | --- | --- | --- | --- | --- | --- | --- | --- | --- | --- | --- |
|  |  |  |  |  | Fe^2+^ | | | Ni^2+^ | | | Cu^2+^ | | | Zn^2+^ | | |
|  |  | **a** | **b** | **c** | **1a** | **1b** | **1c** | **2a** | **2b** | **2c** | **3a** | **3b** | **3c** | **5a** | **5b** | **5c** |
| Lp(1)N_3_ | BD^*^(2) C_2_=O_2_ | 37.19 | 32.38 | 32.40 | 0.59 | 0.61 | - | - | - | - | - | - | - | - | - | - |
| Lp(1)N_2_ | BD^*^(2) C=O_3_ | 59.60 | 36.36 | 39.28 | 0.85 | - | - | - | - | - | - | - | - | - | - | - |
| Lp(2)O_2_ | BD^*^(2) C_2_-N_3_ | 28.57 | 28.50 | 28.45 | 6.88 | 0.51 | 6.59 | 7.97 | 1.28 | 7.71 | 1.99 | 9.28 | 8.31 | 5.94 | 5.77 | 5.73 |
| Lp(2)O_2_ | BD^*^(2) C_1_-C_2_ | 19.86 | 20.98 | 20.95 | 1.24 | 1.33 | 9.38 | 10.37 | 2.34 | 10.05 | 0.26 | 9.16 | 9.46 | 10.62 | 10.52 | 10.49 |
| Lp(2)O_3_ | BD^*^(2) C-N_1_ | 32.01 | 31.43 | 31.37 | 7.45 | 78.88 | 6.71 | 10.91 | 11.31 | 6.56 | 2.48 | 10.01 | 10.09 | 10.57 | 10.04 | 10.06 |
| Lp(2)O_3_ | BD^*^(2) C-N_2_ | 27.29 | 26.82 | 26.79 | 14.95 | 14.21 | 14.04 | 12.66 | 16.51 | 0.70 | - | 10.65 | 10.63 | - | - | - |
|  |  |  |  |  |  |  |  |  |  |  |  |  |  |  |  |  |
|  |  | **A** | **B** | **C** | **6A** | **6B** | **6C** | **7A** | **7B** | **7C** | **8A** | **8B** | **8C** | **10A** | **10B** | **10C** |
|  |  |  |  |  |  |  |  |  |  |  |  |  |  |  |  |  |
| Lp(1)N_3_ | BD^*^(2) C_2_=O_2_ | 1.70 | 4.69 | 1.72 | 17.4 | 12.07 | 18.18 | - | - | - | 0.27 | - | - | - | - | - |
| Lp(1)N_2_ | BD^*^(2) C=O_3_ | 56.11 | 52.98 | 53.68 | - | - | - | - | - | - | - | - | - | - | - | - |
| Lp(2)O_2_ | BD^*^(2) C_2_-N_3_ | 19.86 | 19.85 | 20.00 | 17.28 | 5.81 | 1.44 | 2.29 | 2.74 | 2.52 | 13.82 | 4.61 | 4.73 | 1.85 | 1.88 | 1.90 |
| Lp(2)O_2_ | BD^*^(2) C_1_-C_2_ | 10.94 | 20.33 | 21.60 | 3.56 | 5.00 | 3.10 | 2.56 | 2.80 | 2.76 | 8.71 | 6.85 | 6.88 | 7.02 | 7.16 | 7.19 |
| Lp(2)O_3_ | BD^*^(2) C-N_1_ | 3.27 | 3.26 | 3.37 | 0.59 | 0.61 | 1.17 | 5.77 | 6.23 | 6.51 | 5.93 | 10.04 | 10.16 | 7.59 | 7.35 | 7.43 |
| Lp(2)O_3_ | BD^*^(2) C-N_2_ | 3.24 | 27.01 | 3.11 | 2.04 | 0.98 | 2.62 | 9.76 | 10.43 | 10.64 | - | 10.24 | 10.22 | - | - | - |

| Medium | $\Delta_{f}H_{Sol}^{o}(H^{+})$ | | $\Delta_{f}G_{Sol}^{o}(H^{+})$ | |
| --- | --- | --- | --- | --- |
|  |  | This work |  | This work |
| Gas | 6.197[72] | -6.196 | -26.28[74] | -26.260 |
| Water | -1024.3[72] | -425.863 | - | -458.315 |
| Benzene | -997.3[72] | -238.705 | - | -271.156 |
| DMF | - | -419.691 | - | -452.143 |

**Table S7**: Solvation enthalpies of proton H^+^ ($\Delta_{f}H_{Sol}^{o}(H^{+})$in kJ/mol) and solvation free energy of proton H^+^ ($\Delta_{f}G_{Sol}^{o}(H^{+})$in kJ/mol).

**Fig.1S**: Optimized structures for neutral ADPHT ligand- metal complexes at B3LYP/ Mixed I level.


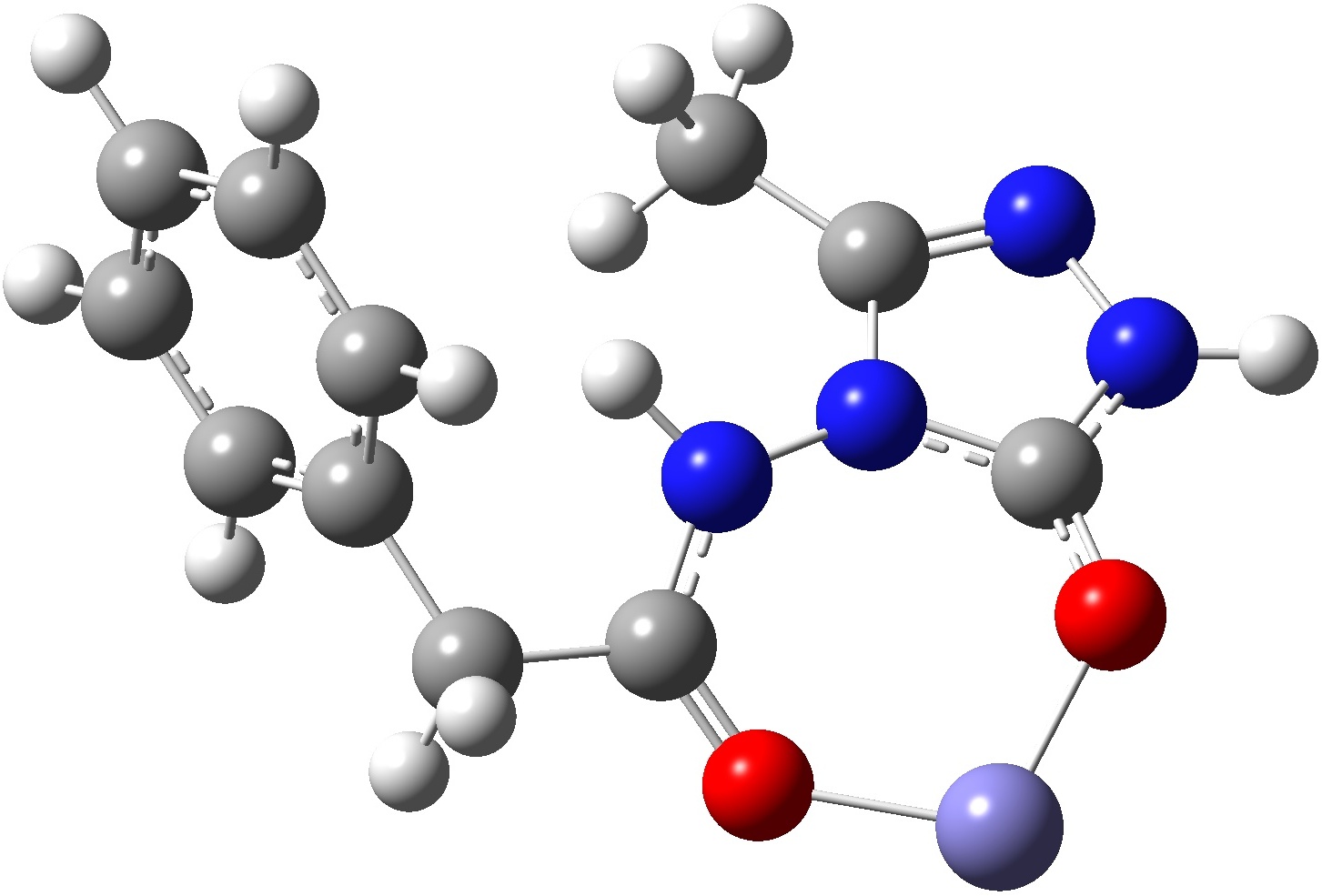


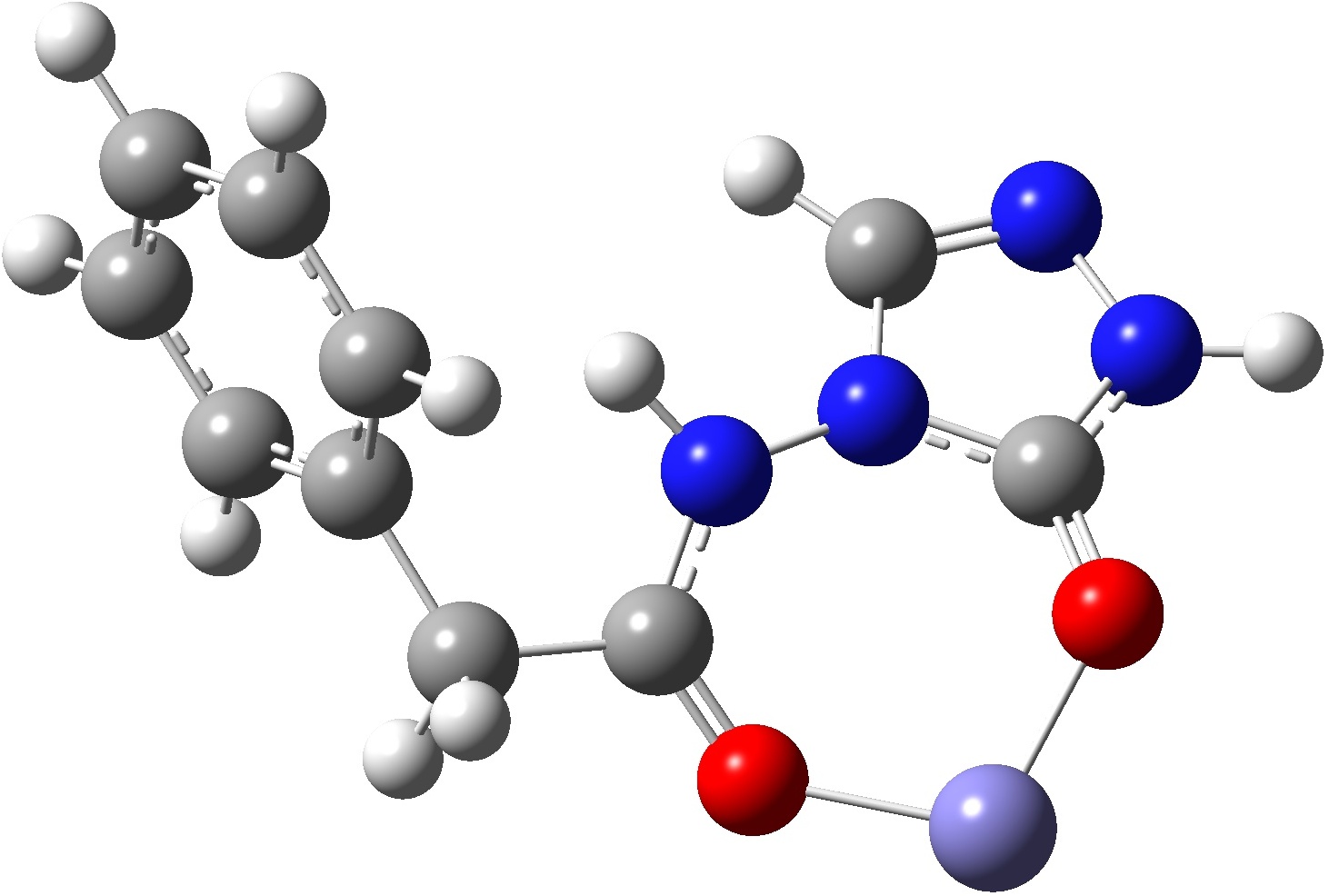


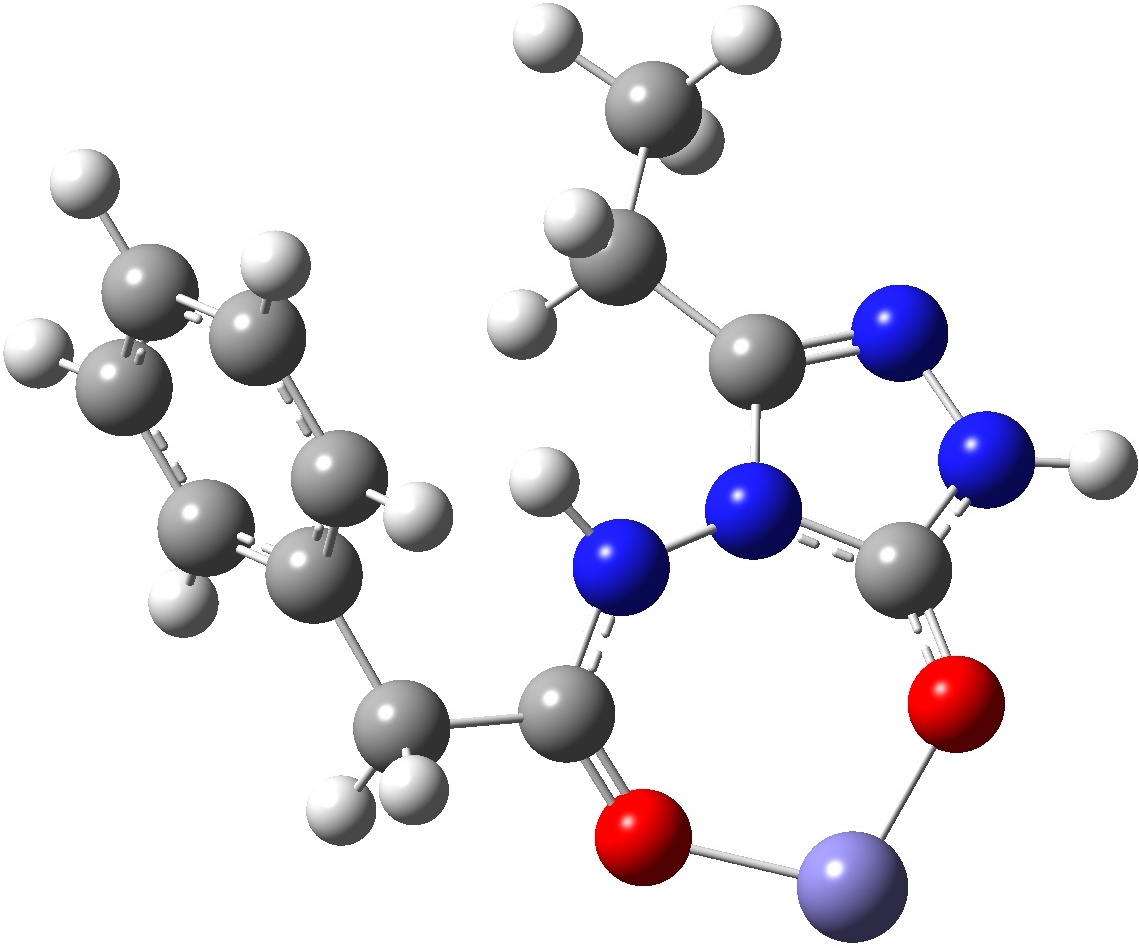


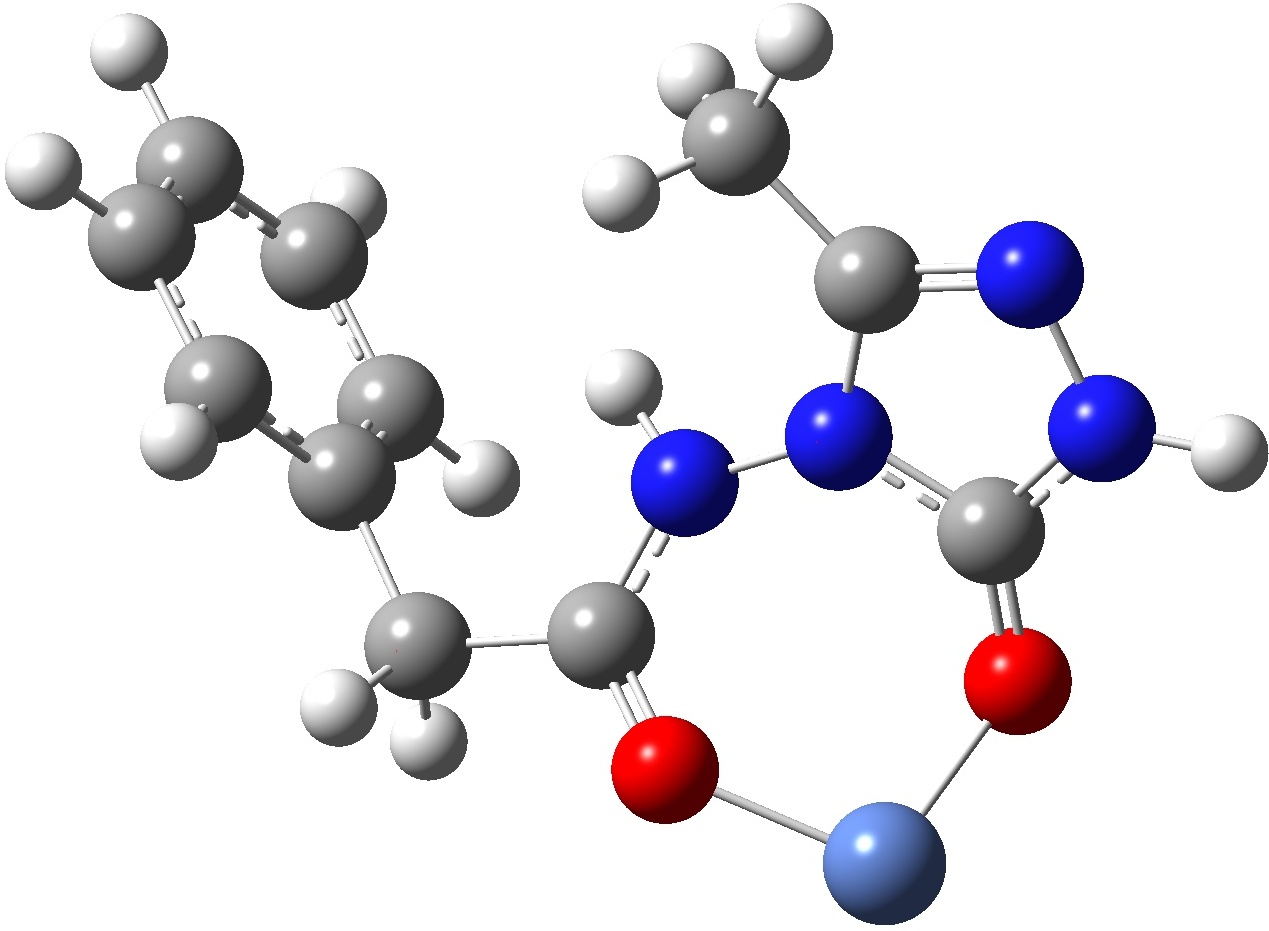


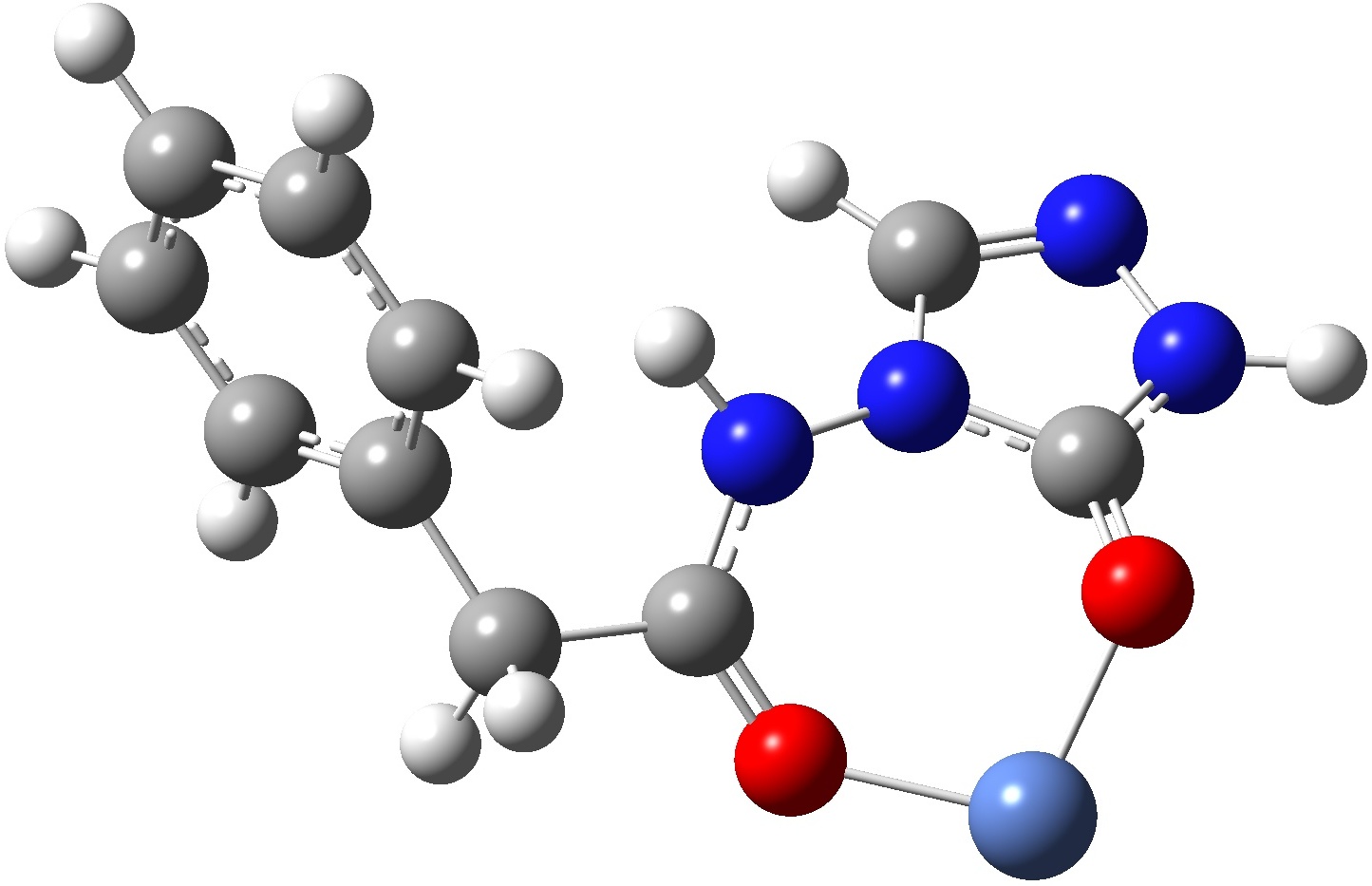


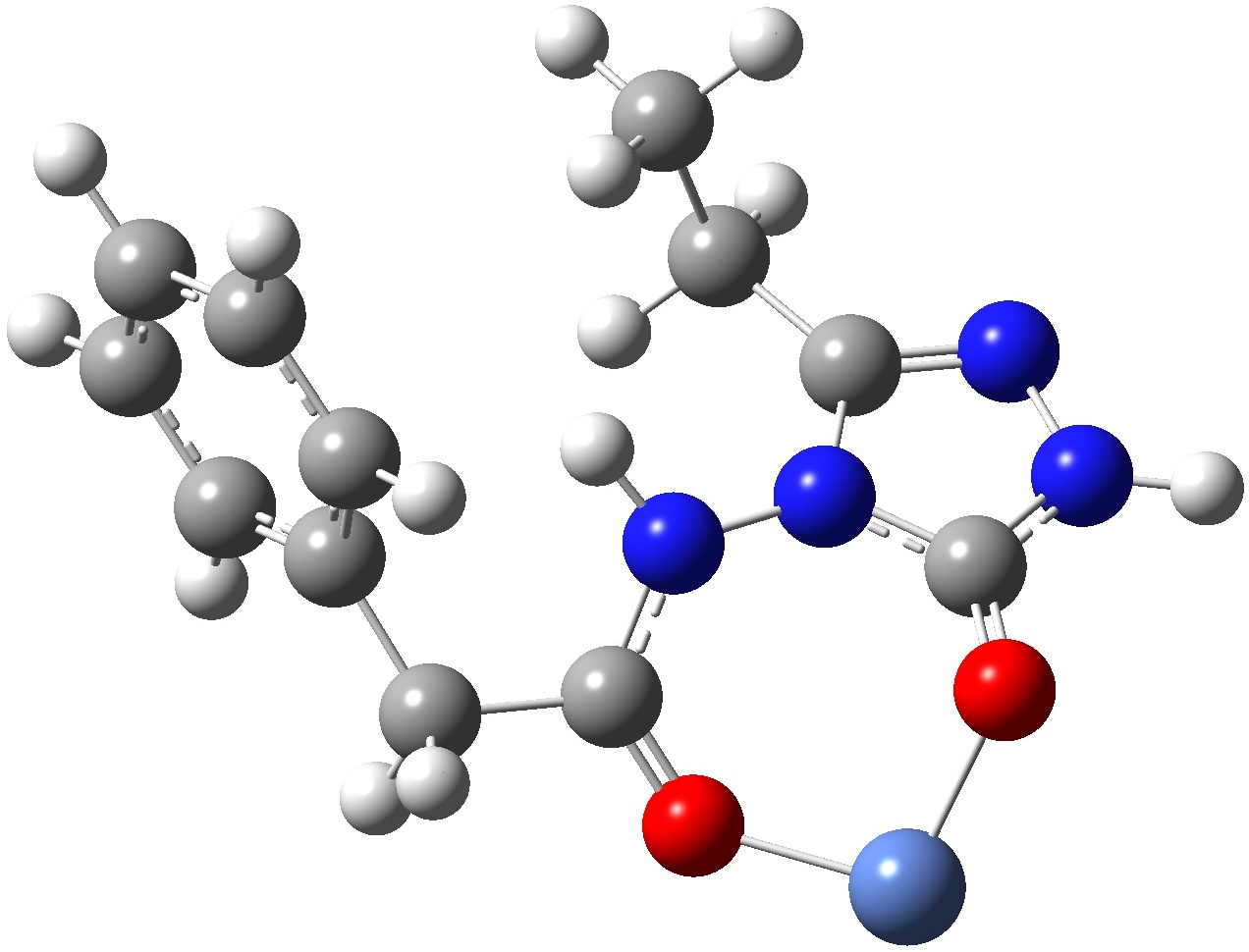


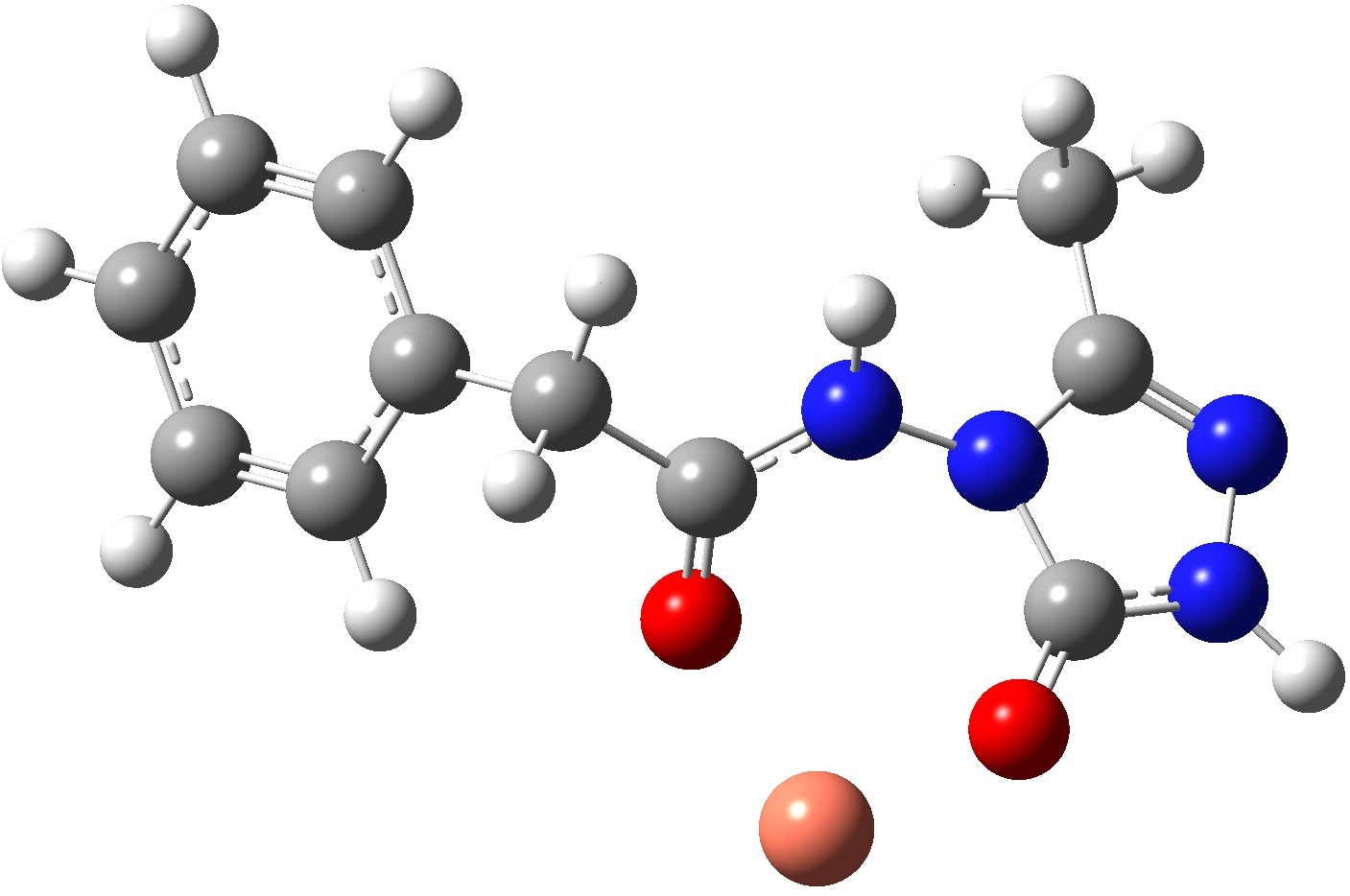


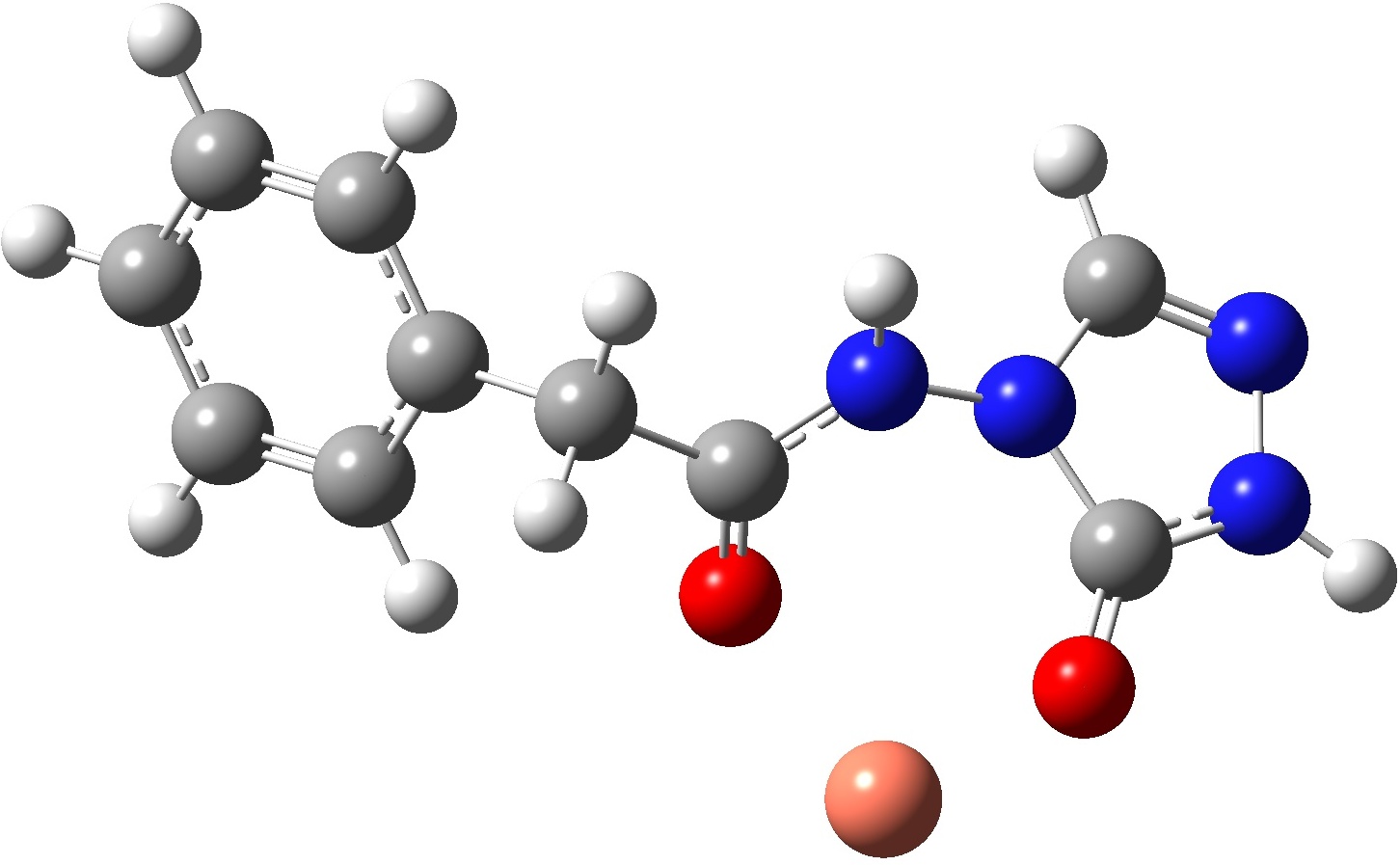


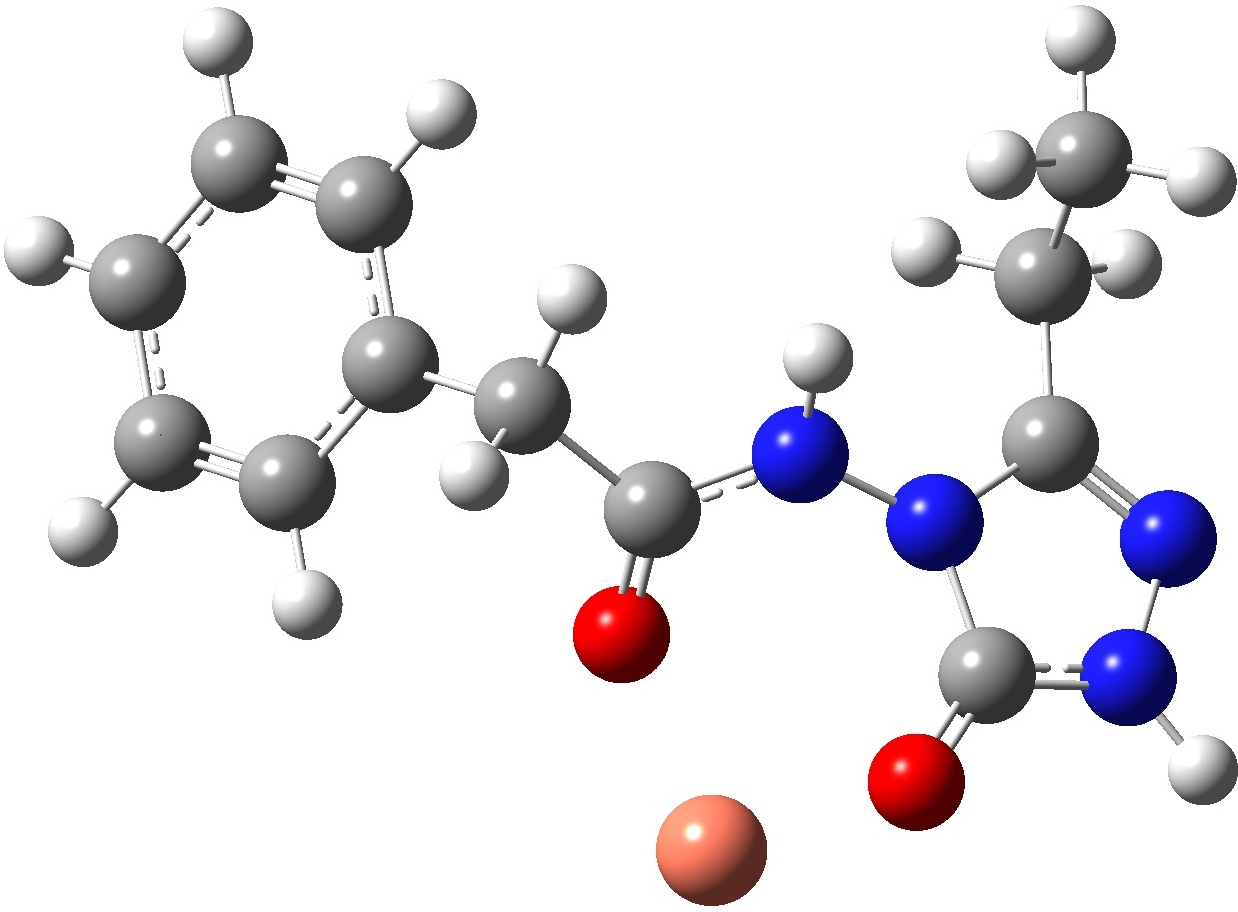


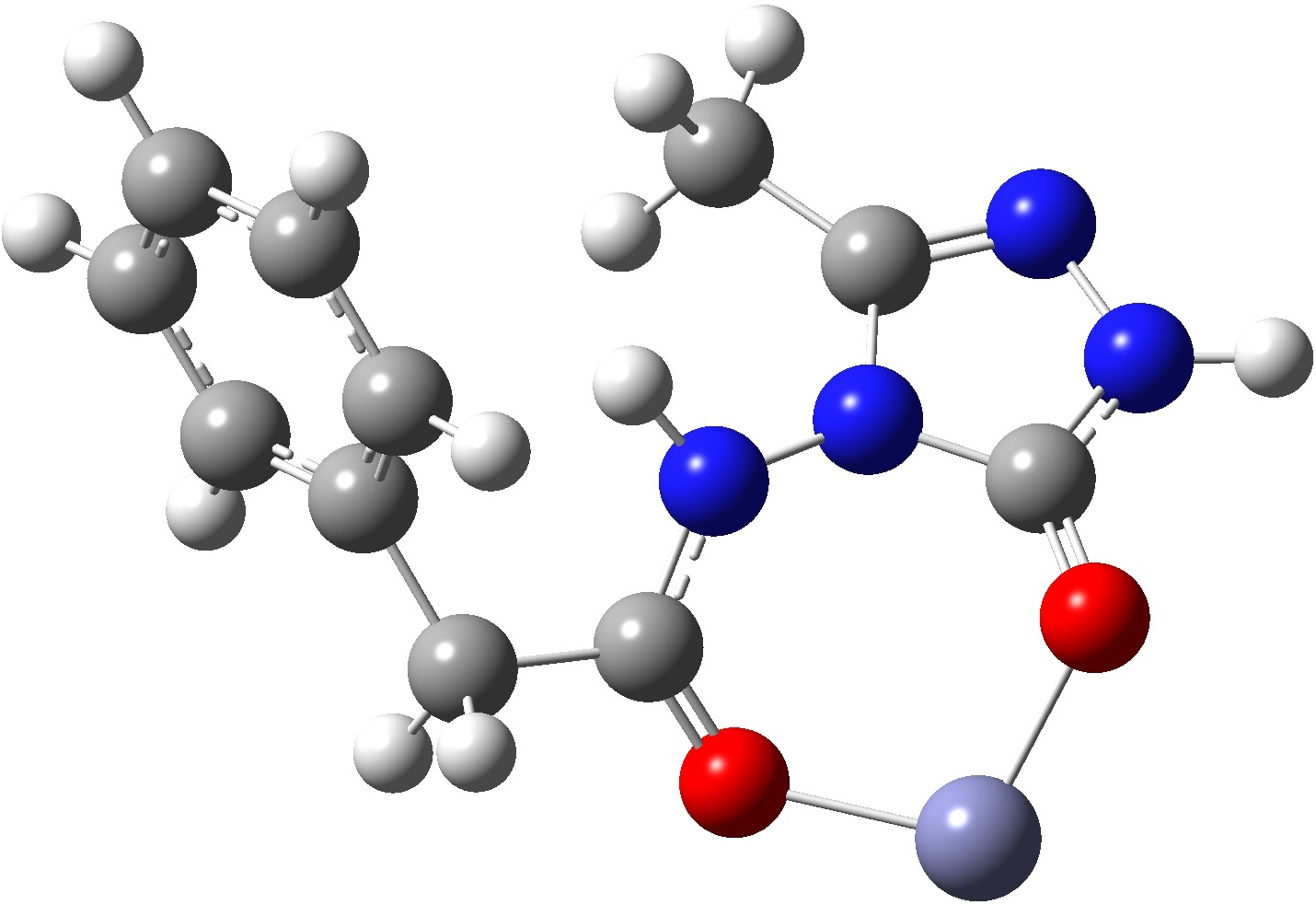


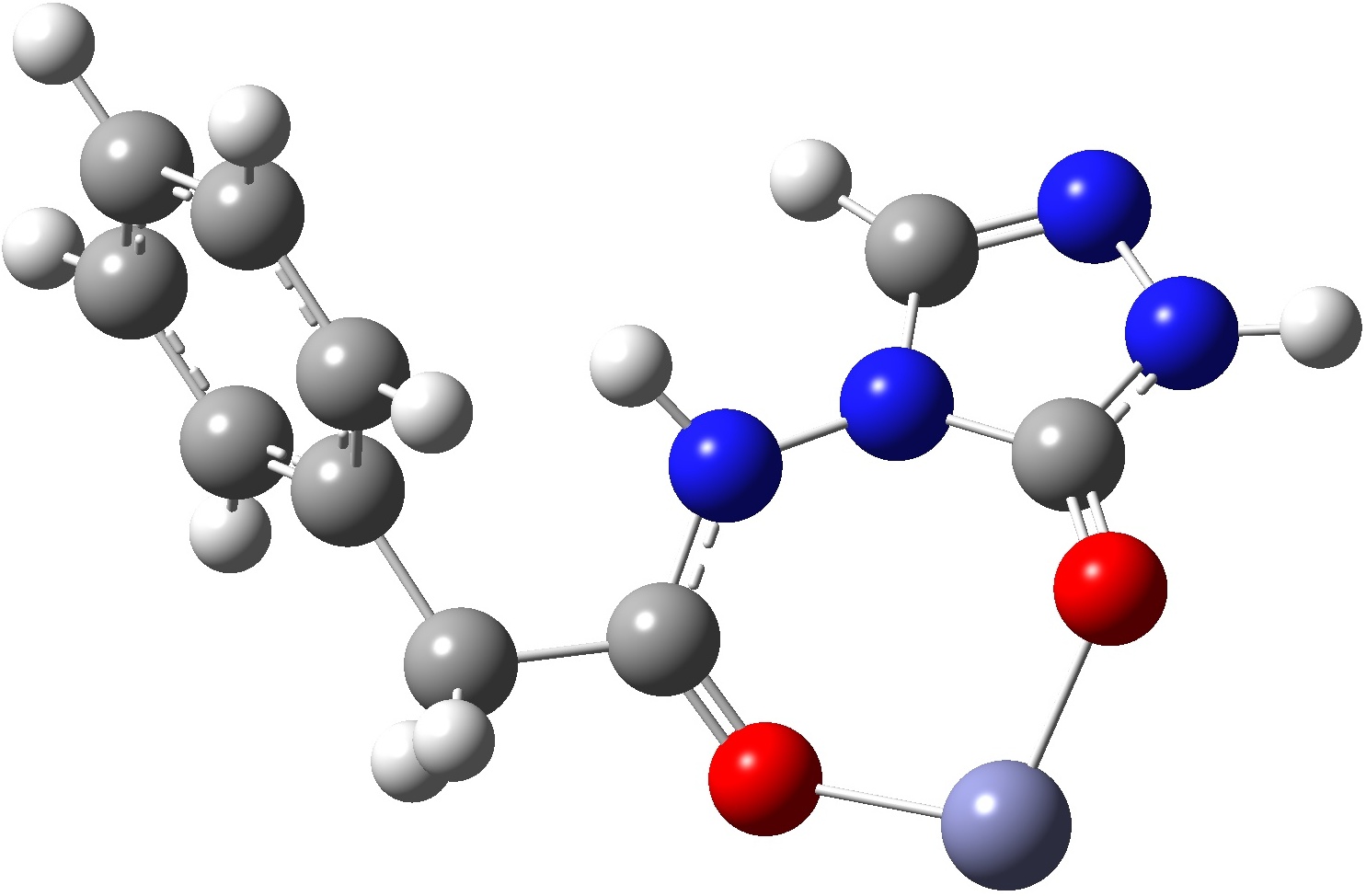


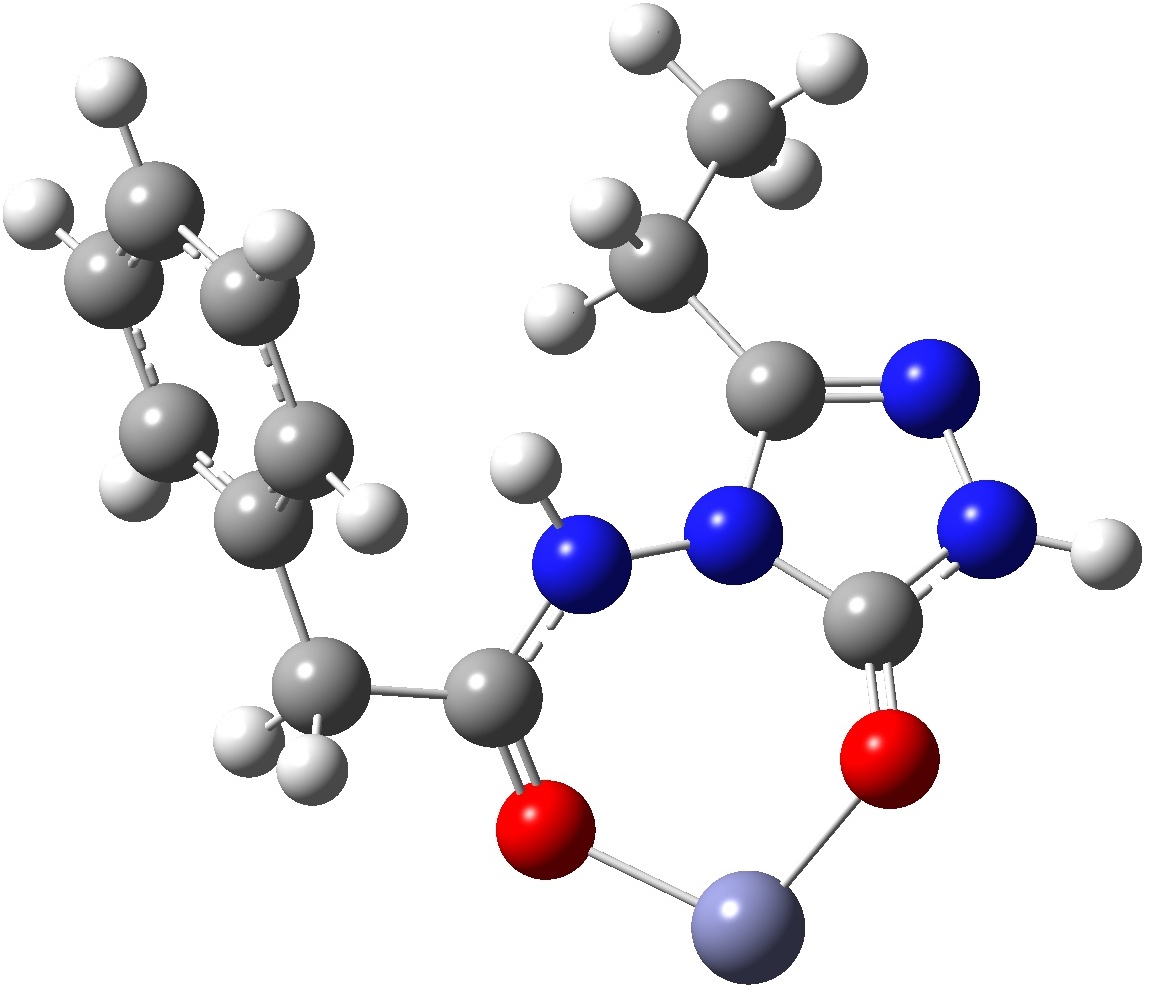


**1a**

**1b**

**1c**

**2a**

**2b**

**2c**

**3a**

**3b**

**3c**

**4a**

**4b**

**4c**

**Fig. 2S** X-H (X = N_2_, N_3_ and C_1_) bond distances for ADPHT ligand- M-ADPHT complexes at B3LYP/Mixed I

**Fig. 3S** C_1_-H_1_ bond distance in different media at B3LYP/Mixed I
